# Biofloc Technology (BFT) in Aquaculture: What Goes Right, What Goes Wrong? A Scientific-Based Snapshot

**DOI:** 10.1155/2024/7496572

**Published:** 2024-01-11

**Authors:** Mohammad Hossein Khanjani, Moslem Sharifinia, Maurício Gustavo Coelho Emerenciano

**Affiliations:** ^1^Department of Fisheries Sciences and Engineering, Faculty of Natural Resources, University of Jiroft, Jiroft, Kerman, Iran; ^2^Shrimp Research Center, Iranian Fisheries Sciences Research Institute, Agricultural Research, Education and Extension Organization (AREEO), Bushehr 75169-89177, Iran; ^3^Commonwealth Scientific and Industrial Research Organization (CSIRO), CSIRO Agriculture and Food, Livestock and Aquaculture Program, Aquaculture Systems Team, Bribie Island Research Centre, Woorim, Australia

## Abstract

Aquaculture is a crucial industry that can help meet the increasing demand for aquatic protein products and provide employment opportunities in coastal areas and beyond. If incorrectly manage, traditional aquaculture methods can have negative impacts on the environment and natural resources, including water pollution and overuse of wild fish stocks as aquafeed ingredients. Biofloc technology (BFT) may offer a promising solution to some of these challenges by promoting a cleaner and sustainable production system. BFT converts waste into bioflocs, which serve as a natural food source for fish and shrimp within the culture system, reducing the need for external inputs, such as feed and chemicals. Moreover, BFT has the potential to improve yields and economic performance while promoting efficient resource utilization, such as water and energy. Despite its numerous advantages, BFT presents several challenges, such as high energy demand, high initial/running costs, waste (effluent, suspended solids, and sludge) management, opportunistic pathogens (*vibrio*) spread, and a lack of understanding of operational/aquatic/microbial dynamics. However, with further training, research, and innovation, these challenges can be overcome, and BFT can become a more widely understood and adopted technique, acting as an effective method for sustainable aquaculture. In summary, BFT offers a cleaner production option that promotes circularity practices while enhancing performance and economic benefits. This technique has the potential to address several challenges faced by the aquaculture industry while ensuring its continued growth and protecting the environment. A more broad BFT adoption can contribute to meeting the increasing demand for aquaculture products while reducing the industry's negative impact on the environment and natural resources. In this context, this review provides an overview of the advantages and challenges of BFT and highlights key technical, biological, and economic aspects to optimize its application, promote further adoption, and overcome the current challenges.

## 1. Introduction

Over the last 30 years, aquaculture has experienced unprecedented growth, currently accounting for more than half of the world's fish needs and playing a crucial role in food security, income generation, and economic development [1, 2]. As a result, aquaculture has emerged as a significant driver of economic growth and poverty reduction in developing countries, with the potential to generate vital income through small-, medium-, and large-scale commercial aquaculture [3–5]. However, to meet the growing demand for safe and high-quality aquatic protein, especially fish and shrimp, aquaculture requires appropriate production systems capable of sustaining higher stocking densities, maintaining acceptable water quality levels, ensuring optimal health and performance, while addressing biosecurity and environmental concerns [6]. In conventional intensive systems, such as earthen ponds, high water exchange regimes are necessary to maintain water quality, and feed inputs are heavily relied upon [7–10]. Unfortunately, the untreated effluent from these systems often contains high levels of pollutants such as nitrogen and phosphorus compounds, leading to poor surrounding environmental conditions [11]. Once the local ecosystem carrying capacity is surpassed, disease outbreaks often occur in densely populated aquaculture areas [12, 13].

Given the challenges associated with conventional aquaculture production systems, biofloc technology (BFT) presents a promising alternative to address critical aspects of water efficiency, environmental impact, and feed costs [14–16]. BFT is known for its high-water efficiency, for instance, (i) reducing water consumption throughout the production cycles by up to 90%, with significant reduction in effluent discharge into the environment; (ii) promoting suitable water quality parameters and microbial profile; (iii) possibility of postharvest mature biofloc water reuse [17–22]. Aquaculture success depends on various biological, technical, and economic factors [23], and BFT is considered as a viable alternative addressing issues such as land use, water consumption, and feeding costs [24]. Depending on the species, feed and feed management, rate of biofloc consumption, and carbohydrate/water supplement costs, BFT might reduce production costs by 33% for green tiger shrimp (*Penaeus semisulcatus*) and 10% for tilapia [25, 26]. Unfortunately, studies assessing the economic aspects of BFT and other production systems are still scarce and create uncertainty when adopting new technologies. The initial response and acceptance of BFT within the broad aquaculture community was slow, but it has increased over the past 2 decades. For instance, lack of successful commercial examples [27], higher running costs (e.g., electricity for aerators and pumping) compared to traditional pond-based systems, lack of knowledge, skilled staff, and relevant information regarding the quality and sensorial attributes of postharvest BFT products, among others, are examples that kept farmers and investors skeptical toward BFT adoption. In addition, seminars and training courses carried-out in key institutions and research centers, especially in Brazil, USA, and Mexico, enabled several professionals to spread BFT knowledge, helping to implement commercial farms worldwide [28]. It is important to highlight that successful examples of BFT adoption at a commercial scale can be found [27, 29]. In this context, this review provides a science-based snapshot of the advantages and challenges of BFT, bringing key management, biological and economic insights that require further attention, aiming to optimize BFT application and overcome the current challenges.

## 2. Understanding Biofloc Technology

Aquatic environmental factors such as temperature, pH, dissolved oxygen, salinity, and nutrient levels can have significant effects on the growth and survival of aquatic organisms [13]. Aquaculture's success is dependent on developing sustainable production systems and management practices that prioritize environmental and biological well-being, as well as ethical and biosecurity considerations. Achieving sustainability in aquaculture means identifying appropriate social, environmental, and financial ratios. In this context, BFT is a microbial-based production system, in which in situ microorganisms present three main roles: (i) water quality maintenance, recycling undesirable toxic N-compounds via key heterotrophic and chemoautotrophic microbial processes; (ii) natural food source provision, decreasing feed conversion ratios (FCR); and (iii) pathogen competition, acting as a natural probiotic [30]. Microorganisms such as bacteria, fungi, algae, and/or protists accumulate due to restricted water exchanges and proper water movement [31]. These proprieties have been shown to minimize environmental impacts [32] with no or minimal effluent to the natural surroundings [33]. To produce microbial aggregates, balancing carbon and nitrogen is necessary, with traditional aquafeeds being the main source of nitrogen and carbon, and affordable products such as sugar cane molasses and grains, as well as inorganic fertilizers, providing the remainder of carbon fraction and other key nutrients [34, 35]. Proteinaceous microbial-based food source containing vitamins, lipids, and carbohydrates is produced in situ [36] and can reduce aquafeed consumption by up to 20% [33, 37, 38]. This positive impact on FCR ratios associated with improved growth and survival [39] is crucial aspects for the system's feasibility, contributing to reduce one of the most significant expenses in aquaculture production [18], while boosting circularity and enhancing green credentials [30]. BFT has been implemented in several countries and regions worldwide, including Vietnam, Brazil, the United States, Iran, Belize, Indonesia, Thailand, Malaysia, Australia, Tahiti, South Korea, Italy, China, as well as Latin and Central American countries [40–43], although levels of adoption and success can vary drastically [44]. In terms of species selection, BFT is most suitable for those that (i) can tolerate a relatively higher levels of N-compounds suspended solids concentrations in the water; (ii) possess morphological adaptations to capture and/or filter the microbial particles; and (iii) support crowding conditions [45]. However, with R&D advances and emerging of hybrid techniques (e.g., BioRAS), the culture environment has been greatly improved with consistent water quality [46], allowing more broad species and culture phases to be explored in BFT (e.g., hatchery *Litopenaeus vannamei* [47], *Oreochromis niloticus* [48]).

Pacific white shrimp and Nile tilapia have been widely used as benchmarks in BFT [18, 49]. However, the successful application of biofloc systems has also been observed in other species such as African catfish (*Clarias gariepinus*) [9], mullet [50], freshwater prawns (*Macrobrachium rosenbergii*) [38], black tiger shrimp (*Penaeus monodon*) [51], banana shrimp (*Penaeus merguiensis*) [52], giant gourami (*Osphronemus goramy*) [53], common carp (*Cyprinus carpio*) [16], rohu (*Labeo rohita*) [54], and bluegill (*Lepomis macrochirus*) [55]. In addition to these individual species, an integrated approach such as polyculture, combining species like catfish and tilapia [56], aquaponics or FLOCponics [57], and integrated multitrophic aquaculture (IMTA) [11] has also shown promising results in recent developments.

## 3. What Goes Right?

### 3.1. Biosecurity, Natural Probiotic Effect, and Immune Enhancer

Closed aquaculture systems are becoming increasingly popular for biosecurity reasons. These systems often provide environmental and social licensing benefits over conventional pond-based extensive and semi-intensive systems [58]. Zero or limited water exchange and reuse of water in closed techniques significantly reduce the chances of introducing external pathogens into the system. In a microbial-based BFT condition, bacterial flocs are typically controlled by cell-to-cell communication via signal molecules in a process called quorum sensing [45, 59]. Quorum sensing regulates the expression of genes encoding for the production of lytic enzymes and toxins in biofilms when a certain cell density is reached [60, 61]. Disrupting cell-to-cell communication in flocs through inactivation of the signaling molecules can reduce the formation of toxic biofilms [61]. Some bacterial communities control virulence factor expression by quorum sensing through natural disruption of cell–cell communications, thereby protecting cultured animals from pathogenic bacterial infections [61, 62]. BFT appears to offer a natural alternative to conventional antibiotics, which may have ecological consequences [61, 63]. Within the biofloc system, accumulation of bacterial storage compound poly *β*-hydroxybutyrate (PHB) normally occurs [64] which possesses antibacterial activity and acts as a preventive curator against vibriosis [65]. In addition, the competition for space and substrate are also mechanisms behind the probiotic effect of BFT, which suppress the multiplication of pathogenic bacteria [44]. Studies show that biofloc has probiotic properties [30, 66, 67] and improves fish/shrimp immunity [68, 69]. Probiotics have gained significant attention in aquaculture as a tool to improve water quality and the performance of farmed animals [70]. The direct use of probiotics in aquaculture systems has been shown to reduce the concentration of toxic nitrogenous compounds, such as ammonia, nitrite, and nitrate, as well as reducing the level of organic matter and pH [71]. Furthermore, probiotics have been shown to reduce the level of pathogenic microorganisms and modulate the microbial community of water and sediment, leading to a more stable and balanced ecosystem [71, 72].

Studies have shown that the use of probiotics in biofloc systems can enhance the production and health of aquatic animals [73, 74]. For example, the use of *Bacillus* sp. in BFT systems has been shown to improve the growth performance and disease resistance of Nile tilapia [74, 75]. Similarly, the use of *Lactobacillus plantarum* in biofloc systems has been shown to improve the growth performance and survival rate of Pacific white shrimp [76]. Thus, probiotics can be used directly in aquaculture and BFT systems to improve water quality by reducing the concentration of toxic nitrogenous compounds, reducing the level of organic matter and pH, reducing the level of pathogenic microorganisms, and modulating the microbial community [70, 73]. Furthermore, the use of probiotics can improve the survival rate and growth performance of farmed animals by enhancing gut health, immune response, disease resistance, and feed utilization efficiency [30, 42]. Several studies have suggested that dietary biofloc has the potential to improve the cellular immune response and antioxidant status of cultured shrimp due to its rich content in natural microorganisms and bioactive compounds [77, 78]. Overall, these findings suggest that BFT can offer nutritional and health benefits for cultured aquatics.

### 3.2. Feed Optimization and Growth Performance

Aquaculture feed is a crucial factor in ensuring the production and profitability of aquatic systems. Typically, feed costs make up a significant portion, ranging from 40% to 60%, of the total production costs in intensive aquaculture operations [79]. Therefore, finding strategies to reduce feed costs becomes essential for improving profitability [45]. Effective feed management, including enhancing the FCR, is key to increasing production efficiency.

One promising approach to reducing feed costs is the implementation of BFT. In BFT systems, microorganisms present in the water are continuously grazed or filtered by shrimp and fish, leading to positive impacts on FCRs [80]. BFT has proven to be successful in reducing feed expenses and alleviating pressure on wild fish stocks by reusing protein found in the feed [33]. Additionally, BFT systems generate microalgae and bacteria with high nutritional value, which can replace up to 100% of the protein derived from fishmeal [81]. By incorporating BFT, up to 29% of the daily food requirement of Pacific white shrimp can be replaced, resulting in improved FCR [82]. The utilization of BFT also benefits producers and consumers by enabling them to consume more animal protein, thus contributing to improved human welfare. Furthermore, studies have shown that in BFT systems, protein utilization is twice as efficient compared to conventional systems, leading to reduced FCR and increased growth rates [39].

In a study conducted by Jatobá et al. [83], it was found that biofloc culture with a density of 250 shrimp/m^3^ resulted in higher yield, reduced protein usage, and lower feed costs compared to traditional pond-based culture with a density of 15 shrimp/m^2^. Similarly, Gaona et al. [84] reported that the FCR for *L. vannamei* decreased from 1.49 in conventional water-based culture to 1.23 in BFT. Deb et al. [85] also found that the FCR for *L. rohita* decreased from 2.78 in conventional water-based culture to 1.69 in BFT. Haraz et al. [86] compared the FCR of Nile tilapia in different systems and reported values of 1.89, 1.80, 1.54, and 1.41 in conventional water-based culture, conventional water-based culture with *Bacillus* sp. probiotic, BFT with a carbon-to-nitrogen ratio of 10, and BFT with added *Bacillus* sp., respectively. Furthermore, BFT has been shown to enable the reduction of dietary protein in some cases. For example, studies by Shao et al. [87] and Olier et al. [88] found that BFT allowed for a decrease in dietary protein for shrimp (*L. vannamei*). Similar results were observed for Nile tilapia (*O. niloticus*) in studies conducted by Azim and Little [89], Tubin et al. [90], and Durigon et al. [91], as well as for pacu fish (*Piaractus mesopotamicus*) in a study by Sgnaulin et al. [92].

There are several strategies for optimizing feed and feeding in aquaculture. One approach is the use of alternative feed ingredients, such as pizzeria by-products and insect meals, under biofloc conditions [93, 94]. Another strategy involves feed deprivation [95]. Additionally, premium protein ingredients can be replaced with “biofloc meal” [96, 97]. This meal can be produced in bioreactors or collected directly from shrimp/fish ponds and tanks [28].  Table 1 presents some of the studies conducted on the substitution of feed with biofloc. Numerous studies have been conducted on the substitution of feed with biofloc, with findings suggesting that feed input can be reduced by up to 50% by promoting bioflocs within culture units [30, 97, 107]. For example, a study by Rani et al. [106] found that microfloc meal (MFM) can effectively replace fishmeal as a partial protein source for *Cirrihinus mrigala*, without compromising performance. This suggests that MFM could be a sustainable protein source, reducing reliance on fishmeal, and alleviating pressure on natural fish stocks. Moreover, the use of MFM could help address the issue of effluent disposal in aquaculture operations. Overall, finding renewable and sustainable alternatives to fishmeal is an environmentally friendly and socially responsible strategy for achieving sustainability in the aquaculture sector. The results of this study highlight the potential of MFM as a viable alternative protein source for fish feeds, contributing to the development of a more sustainable aquaculture industry.

Enhancing the growth performance and survival rates are crucial to reduce production costs and optimizing profits [108]. The outcomes of BFT regarding different performance measures such as survival rate, production, stocking density, initial and final weight, and rearing period are demonstrated in Table 2. For instance, in study by Khanjani et al. [125] found that the survival rate of juvenile tilapia in biofloc treatments was significantly higher (98.2%) than in clear water-based (95.35%). Similarly, higher survival rate was found in biofloc-based *L. vannamei* culture versus clear water (CW) exchange systems [39, 126]. In terms of growth, aquatic species reared in BFT generally displayed superior performance compared to conventional CW or pond-based systems. Azim and Little [89] found the net tilapia production was 45% higher in the BFT tanks than in the control tanks confirming the utilization of bioflocs by fish as food source. In study by Ray et al. [127] observed shrimp (*L. vannamei*) production in biofloc systems increase by a remarkable 41% compared to conventional CW systems. The microorganism community also enhances the digestive system, leading to growth increases of up to 15% in *L. vannamei* and reductions in FCRs of up to 40% [39]. The profitability and return on investment in aquaculture are greatly influenced by biological parameters such as survival rate, growth performance, and stocking density [45, 128, 129]. Improving growth performance and survival rates is crucial for reducing production costs and maximizing profits. Posadas and Hanson [108] developed a set of financial and economic performance measures that incorporate biological parameters, capital costs, variable costs, and shrimp prices. These measures include yearly cash flows, net present values, and internal rates of return. Survival rate plays a critical role in cost returns and profitability, as it directly impacts total production [129]. The profitability of shrimp and fish farming depends on three key factors: production level, production cost, and the market price of shrimp and fish [130]. Market demand also needs to be considered by farmers to ensure the feasibility of their operations. According to Poersch et al. [79], the long cycle (LC) treatment was found to be more profitable than other treatments, primarily due to a higher final average weight, productivity, selling price, and similar fixed costs. Even under subtropical conditions, the use of one or two crops per year does not significantly affect shrimp productivity in lined ponds, and the LC treatment proves to be more profitable due to the larger size of the shrimp produced. In Poersch et al.'s [79] study, despite a lower survival rate of 68%, the LC treatment resulted in higher gross income. This was attributed to the increase in productivity resulting from a larger average weight (26 g), which commands higher market prices.

Aquatic animal survival rates greatly impact total production. In the laboratory, tilapia juveniles have been successfully produced in BFT at an initial density of 1,250 fish/m^3^. The survival rate is 96%, and the biomass ranges from 15.12 [19] to 37 kg/m^3^ [131]. By using biofloc as a supplementary food, it is possible to decrease the amount of dietary protein needed for tilapia juveniles in BFT from 36% to 28% of their body weight. This reduction does not affect the animals' performance [132]. Since feed cost is a significant expense, reducing protein levels is crucial for maximizing profitability.

### 3.3. Environmental Attributes and Economics

Aquaculture effluent is a major source of organic carbon, suspended solids, phosphates, nitrogenous species (nitrates, nitrites, and ammonia), chemical oxygen demand, and biological oxygen demand. This poses a serious threat to aquatic ecosystems worldwide, as it can negatively impact surrounding waters and groundwater [133]. However, according to a recent study by Jones et al. [134], global wastewater production is estimated to be 359.4 × 10^9^ m^3^/year. Of this, 63% (225.6 × 10^9^ m^3^/year) is collected, and 52% (188.1 × 10^9^ m^3^/year) is treated. It is estimated that 48% of global wastewater production is released untreated into the environment, a much lower figure than previous estimates of about 80%. The study also found that approximately 40.7 × 10^9^ m^3^/year of treated wastewater is intentionally reused. The per capita production, collection, and treatment of wastewater vary significantly across different geographic regions and levels of economic development. For instance, just over 16% of the global population in high-income countries produces 41% of global wastewater. The Middle East, North Africa, and Western Europe have the highest rates of treated-wastewater reuse, at 15% and 16%, respectively, despite comprising only 5.8% and 5.7% of the global population [134]. Environmental pollution costs have been evaluated using the material balance method and shadow price method. The shadow price refers to the price at which various economic resources should be obtained under optimal allocation of production. Natural resources are priced based on the marginal productivity of resource shadow prices. Scarce resources command a higher price than those that are abundant. In the case of BFT, water exchange is limited, thereby eliminating waste and associated environmental costs [113]. The use of BFT can significantly reduce water consumption in aquaculture, as it uses minimal or zero water exchange during production [17]. For example, *M. rosenbergii* and *O. niloticus* in BFT were reported to consume 6.8 and 0.071 m^3^ of water per kg of production, respectively [135, 136]. The water consumption of *L. vannamei* grown in saltwater using BFT is also reported to decrease (0.098–0.169 m^3^ of water per kg of production) [20]. Compared to traditional freshwater aquaculture, which uses 16.9 m^3^ of water per kg, BFT farming techniques are both environmentally friendly and increase productivity [137]. BFT has been shown to reduce water consumption even further when the same water is reused in multiple culture cycles [20]. In addition to reducing water consumption, BFT is more productive than traditional fishponds and requires a smaller area of production [116, 138]. This makes it a more convenient and closer-to-urban-centers option.

Compared to conventional water treatment technologies in aquaculture production, BFT provides an economic advantage and is also viewed as a sustainable water treatment technique [33, 135]. BFT requires less maintenance, produces fewer secondary pollutants, and can reduce water use costs by 30% [33, 34]. Furthermore, BFT can be used in regions with water restrictions, as it reduces the water demand of juvenile tilapia by up to 12 times [139]. Therefore, the use of BFT could be an ideal technology for aquaculture far from water bodies [115].  Table 3 provides a comparison of the water consumption rate between the biofloc system and the conventional system. Understanding the water usage in aquaculture systems is crucial in terms of minimizing the environmental impact and optimizing resource utilization. Therefore, the results presented in Table 3 can be valuable for developing sustainable aquaculture practices that conserve water resources and reduce the ecological footprint of aquaculture operations. Moreover, BFT can maintain water quality at adequate levels that support high productivity (>5 k and >20 kg/m^3^ for shrimp and fish, respectively) and survival rates (e.g., >90%, 92%, 101%). BFT offers a solution to water quality issues in aquaculture by utilizing minimal water exchange and bacterial activity to break down residual organic matter [17, 141]. Additionally, the water used after shrimp and fish harvest can be reused multiple times as a microbial inoculum for subsequent cycles. This not only enhances water quality and performance but also reduces water consumption and waste generation [20, 21]. Studies have demonstrated that applying BFT to catfish production can reduce water consumption by up to 14 times [142]. Overall, by maintaining water quality, minimizing water usage and waste generation, and improving feed efficiency to reduce costs, BFT proves to be economically feasible, environmentally friendly, and socially accepted [143–146]. BFT allows for intensive and superintensive shrimp production in smaller areas with high stocking densities ranging from 100 to 450 shrimp/m^3^ [147]. The high stocking density of BFT requires constant monitoring and maintenance of water quality parameters [148–150]. Despite the significant investment required to implement and operate BFT systems [151], it offers environmental, sanitary, and economic advantages [111, 117, 152–154]. Stocking density has a direct impact on production and profitability [120, 155, 156]. BFT systems allow for greater production with smaller cultivation areas and improve the efficiency of production factors, thus increasing profitability [115, 148]. In a study conducted by Nazarpour and Mohammadiazarm [155], they examined the impact of various stocking densities of common carp in the biofloc system. The results showed that the fish exhibited optimal performance when stocked at densities of up to 250 fish/m^3^ in the biofloc system. Mauladani et al. [157] found that a net profit of US$ 13.81/m^2^ was achieved using nanobubbles in a superintensive BFT production system with a density of 400 shrimp/m^2^ and an average final weight of 10.10 g. According to Browdy et al. [158], profitability can be increased by 57% and 45% through a 20% increase in stocking density and growth rate, respectively. Consequently, the aquaculture industry has experienced growth due to intensified practices, species diversification, and the implementation of innovative technologies [159]. Table 2 displays the stocking densities used in various aquaculture systems, revealing that higher stocking densities can result in increased production rates in biofloc systems. This finding underscores the importance of stocking density management in optimizing production efficiency in biofloc systems. By carefully managing stocking density, aquaculture practitioners can enhance production rates, while minimizing the ecological footprint of their operations. Biofloc systems have become more profitable due to their reduced culture period and higher growth and survival rates [38, 89].  Table 4 presents a summary of various economic studies that have been conducted to assess the costs of implementing biofloc systems in aquaculture. The studies have explored different cost components, such as design, labor, energy consumption, feed, larvae, and fingerlings. The findings of these studies can provide valuable insights into the economic feasibility of adopting BFT in aquaculture operations. This information can help aquaculture practitioners make informed decisions about whether to implement biofloc systems and how to optimize their operations to maximize economic benefits while minimizing costs.

To support sustainable aquaculture developments in the future, environmental costs need to be considered. Currently, the government is primarily responsible for these costs, not farmers. However, sustainable aquaculture and environmental protection may be undertaken by farmers themselves in the future. Environmental costs include water resource costs, feed costs, and pollution costs [162, 163]. The environment and feed supply are two of the main factors that can affect the growth and development of the aquaculture industry [17, 164]. Therefore, farmers aim to reduce production costs, increase profitability, and minimize environmental impact.

### 3.4. Reproduction Performance and Carcass Quality

Studies have shown that BFT can enhance the nutritional quality, reproductive performance, and early larval development of shrimp and fish [165–168]. Biofloc is a significant source of dietary lipids, including phospholipids and essential fatty acids, that are important for reproduction and embryonic and larval development in various species of aquatic organisms, such as *Litopenaeus stylirostris* [169], *F. duorarum* [170], *L. vannamei* [170], *F. brasiliensis* [171], *O. niloticus* [168, 172], *C. carpio* [173], and red tilapia [174]. The nutritional status of the female shrimp is an important factor that can influence reproductive performance and embryonic development [175, 176]. Biofloc can contribute to the nutrition of shrimp and fish by providing a variety of nutrients, including protein, lipids, fatty acids, and vitamin C [33, 165, 177]. Lipids, such as phospholipids and essential fatty acids, are believed to be crucial nutritional factors for the reproductive process, egg-hatching rate, and larval survival of shrimp and fish [178, 179].

Broodstock reared in BFT systems has been found to have improved health and survival rates, which may contribute to better reproductive performance [169, 180]. In particular, broodstock from biofloc systems has lower oxidized glutathione (GSSG)/total glutathione (GSH) ratios and better antioxidant status, marked by higher concentrations of GSH and total antioxidant status. This improved health and survival may be linked to better resistance to handling stress caused by fishing, transfer to hatchery, and eyestalk ablation [181]. Overall, BFT has the potential to improve aquaculture sustainability and productivity by enhancing the nutritional quality and health of shrimp and other aquatic organisms. Various factors, such as nutrition, environmental conditions, and farming systems, can influence the quality of fish meat [182]. The biofloc system, which encompasses these factors, can have an impact on the quality of fish produced. One key concern in this farming system is the quality of the fish. The limited water exchange and high bacterial load in the biofloc system, along with the consumption of bioflocs by fish, can have a significant effect on the sensory quality and characteristics of the fish. However, there is limited information available on the quality characteristics of fish fillets raised in this system. In a study by Bakhshi et al. [183] that focused on the quality of common carp meat in the biofloc system, four treatments were examined. These treatments included a control group without bioflocs, and three biofloc treatments using different carbon sources (molasses, sugar, and starch). The molasses biofloc treatment showed a more desirable skin reddening index compared to the control group. Najdegerami et al. [184] suggested that this effect on skin color may be due to the pigments present in the bioflocs.

Additionally, the research conducted by Abdollahi Khazaghi et al. [185] revealed that the reddening of fish meat is closely linked to the existence of Fe^+3^ ions and the regulation of their oxidation process. When probiotics are included in the fish's diet, they can effectively mitigate the degradation of the red color by controlling the oxidation reactions. Moreover, probiotics have the ability to enhance the intensity of tissue redness by oxidizing heme compounds and binding with essential amino acids such as lysine, cysteine, methionine, and tryptophan. In general, the implementation of the biofloc system holds promise for improving the quality of fish meat due to various contributing factors. These include the presence of pigments in bioflocs and the utilization of probiotics that not only regulate oxidation reactions but also intensify tissue redness [183].

## 4. Biofloc: What Goes Wrong?

Implementing BFT systems can be challenging and expensive due to inappropriate water quality management, lack of skilled staff, inappropriate system design, the higher installation and operating costs associated with intensive aeration, and the removal of suspended solids in the water column [18, 117, 186].

### 4.1. Inappropriate Water Quality Management

Water quality parameters such as pH, alkalinity, TSS, and and N-NO_3_ are directly related to the conditions of the BFT system, in which the formation, aggregation, and metabolism of microbial communities, especially nitrifying autotrophic and heterotrophic bacteria, consume alkalinity, reducing pH, increasing TSS, and transforming ammonium into nitrate, due to the nitrification process [45, 187]. Lack of proper toxic N-compounds management (high TAN and NO_2_ due to improper C : N management or nitrifying bacteria management), overuse of organic carbon and lack of “sludge removal”, can lead to solids accumulation and pathogenic bacterial spread. In experimental scale [188] and in commercial scale [29] in Vietnam demonstrated if external carbon and C : N (e.g., sugar cane molasses) is proper managed, biofloc can outcompete pathogenic bacteria. The proper C : N stoichiometric calculations, solids/sludge management, and microbial ecology knowledge are crucial steps to avoid pathogens issues.

#### 4.1.1. Solids Disposal

Similar to RAS routine management, the solids in BFT from routine sludge removal using “toilets” or from mechanical filters (e.g., clarifiers removing the excess of suspended particles water column) need for a proper solids disposal. Additionally, the ability of cultured fish to tolerate high suspended solid concentration must be considered, as this can adversely affect the growth of certain fish species. A compilation of various studies that have investigated the levels of total suspended solids in the biofloc system is demonstrated in Table 5. High TSS concentrations can cause skin irritations, fin erosion, blockage of the opercula cavity, gas diffusion inhibition, nitrogen compound excretion, and changes in ion exchange [188, 193]. However, BFT systems are typically operated at TSS concentrations below 1,000 mg/L and most often less than 500 mg/L [18]. The negative impacts on water quality parameters can be reduced by using low TSS concentrations, starting the culture at approximately 100 mg/L, and reducing variations over time. Respiratory rates can increase in situations where O_2_ uptake is not efficient, leading to a CO_2_/HCO_3_ imbalance in the blood [194–196], which may explain the respiratory alkalosis observed in fish in the BFT system [190]. Biofloc farming presents several challenges that require careful consideration before starting. Proper training is essential, and maintaining the size and temperature of water tanks, as well as ensuring a constant oxygen supply to pond water, are critical [141]. The size and breed of fish/shrimp also matter, and density in water must not exceed recommended levels [45]. It is advisable to keep biofloc farms outside of sheds, although they can be built under a roof shelter with an open side wall. Checking the percentage of minerals in water is crucial, and natural light is important for the growth of fish/shrimp cultured in biofloc [89]. Additionally, boundaries of biofloc ponds must be air and temperature resistant, and fish breeds must have mutual understanding without fighting, similar to mixed cropping of plants [45]. Despite these challenges, biofloc farming can be a profitable and sustainable practice when properly managed. One of the major challenges in biofloc farming is maintaining adequate alkalinity levels. Alkalinity is constantly depleted by reactions with acid added to water, particularly in intensive biofloc systems where nitrifying bacteria activity is responsible for most alkalinity losses [18, 33]. Once alkalinity is depleted, pH can drop steeply, inhibiting bacterial function and limiting fish appetite and feeding response. Alkalinity should be kept between 100 and 150 mg/L as CaCO_3_ by regular additions of sodium bicarbonate [18].

Another challenge of the biofloc system, when water is reused for multiple consecutive crops (e.g., inland *L. vannamei* farming), (i) nitrate and phosphate accumulation and (ii) the mineral profile can be affected and need ionic profile adjustments. More information is available regarding key macrominerals (e.g., K, Mg, and Ca), but it is scarce the literature regarding micro and trace elements in long-term reuse conditions [197].

### 4.2. Inappropriate System Design

High-density rearing in aquaculture requires waste treatment infrastructure, and biofloc systems are a type of waste treatment system. These systems were developed to prevent disease introduction and are used in closed and intensive shrimp/fish farming. Superintensive shrimp culture systems have specific engineering and management criteria that are still being explored. There are two types of biofloc systems: those exposed to natural light (green water) and those without exposure to natural light (brown water) [198]. Most biofloc systems in commercial use are green water, while brown-water systems are operated solely by bacterial processes. Two primary BFT systems for shrimp culture are in situ systems (where biofloc form in the culture pond/tank) and ex situ systems (where effluent waters are diverted into a biological reactor). In situ systems have benefits such as assimilating ammonia into microbial proteins and providing nutrition directly to the shrimp, but they lack control over nutritional profiles and have a high oxygen demand. Ex situ systems offer better control of floc nutritional profiles and separation of oxygen demand between floc and shrimp [28]. To construct and prepare BFT ponds, it is necessary to conduct a detailed study. Attention should be given to the shape, size, depth, pond lining, and central drainage system in the construction of the pond. The classical design of BFT ponds is typically round, with aerators creating radial water flow. Alternatively, square or rectangular ponds can be used, with water flow also being radial or parallel to the pond dykes. In both cases, corners are usually rounded or cut to minimize stagnant areas [45]. Round ponds are commonly used for small ponds in hatcheries and some production units, while larger ponds are often rectangular or similar in shape due to the challenges of construction and land utilization. Intensive ponds should not be too large due to difficulties in controlling large volumes of water and harvesting high biomass. Holding dense fish or shrimp populations in very large reservoirs also presents higher risks. The typical size range for intensive ponds is between 100 and 1,000 m^2^, while for intensive BFT shrimp ponds, it is 1,000–20,000 m^2^ (0.1–2 ha). The depth of ponds is usually 1–2 m. Deep ponds have the advantage of high heat buffering capacity, which helps regulate temperature fluctuations. They also minimize contact between surface water and anaerobic conditions at the pond bottom, while providing a deeper water column for feeding and biological processes. However, constructing deeper ponds requires higher investment and can pose challenges for drainage and harvesting, especially in areas with limited gradient to the drainage base. A recent concept in aquaculture is the use of shrimp toilets or central drains. Aquaculturists are establishing these pits or drains at the center of the culture pond, utilizing around 5%–7% of the total surface area. The ideal pond size for a shrimp toilet is between 1,000 and 5,000 m^2^. The establishment typically includes a concrete cement structure with a smooth slope leading to a small well of about 2–3 feet depth at the center. The smooth and sloped surface allows for the fast movement of waste toward the central pit, reducing the water requirement for waste removal. Intensive aeration helps to continuously move waste materials into the well. The waste can then be removed using a siphoning motor or submersible or floating pump (with a power of about 2 hp) on a weekly basis to prevent the accumulation of sludge. In general, standardizing methods, techniques, and equipment for pond construction, stoking management, and harvesting in BFT aquaculture systems are essential for proper design.

### 4.3. Lack of Skilled Staff

In 2020, the total production of fisheries and aquaculture reached a record high of 214 million tonnes, with 178 million tonnes coming from aquatic animals and 36 million tonnes from algae. This growth was primarily driven by the expansion of aquaculture, particularly in Asia. The amount of seafood available for human consumption (excluding algae) was 20.2 kg per capita, which is more than double the average of 9.9 kg per capita in the 1960s [199]. The primary sector alone employed around 58.5 million people, and when including workers in subsistence and secondary sectors, as well as their dependents, it is estimated that approximately 600 million livelihoods depend at least partially on fisheries and aquaculture [199]. Given the large number of people involved in the industry, there is a constant need for qualified personnel, especially in new aquaculture systems. It is crucial to train qualified individuals to improve and address sudden failures in BFT systems, as the controlled management of bacteria and cultured organisms is essential. Various countries have implemented training programs to develop qualified staff in different disciplines. For example, a marine aquaculture project in Morocco supported the training of qualified personnel at a dedicated center [200]. Education plays a vital role in enhancing the skills and qualifications of personnel in aquaculture. In this regard, e-learning education can be a valuable alternative, especially when it does not disrupt the regular work of personnel [201].

### 4.4. High Energy Demand, Initial/Implementation Costs

Floc formation in BFT systems occurs due to continuous aeration and water column agitation. Continuous and strong aeration has several effects on BFT systems, including providing oxygen to cultured organisms, preventing negative impacts of high stock densities, ensuring homogeneous oxygen distribution, agitating the water column, oxygenating the sediment, and supporting nitrification by providing oxygen to the microbial community [17, 18]. However, continuous and strong aeration can lead to high operating costs. Different aeration equipment, such as propellers, aero tubes, diffusers, air stones, paddlewheels, nozzles, and vertical pump aerators, can be used to minimize energy costs [202]. The turbulence generated by aeration units affects floc collection and breaking. Studies have investigated the effects of continuous and intermittent aeration on BFT systems. One study found no significant differences in nitrogenous compounds and biofloc content between continuous and intermittent aeration groups, suggesting that intermittent aeration has potential for reducing energy costs [203]. Another study examined the presence of nitrogenous compounds in uncultured media with different aeration rates, finding that nitrate concentrations were higher in the nonaerated group [204]. In a comparison of different aeration units, aero tubes resulted in higher water quality, biofloc volume, and shrimp biomass due to more homogeneous mixing and circular water current [205]. The use of diffused air blowers showed the best performance in the nitrification process and resulted in the highest productivity. Additionally, microbubble aeration was found to improve water quality and increase shrimp growth efficiency [206].

Creating optimal conditions for aeration, which is typically associated with high energy costs, can reduce operating expenses and increase product yield in BFT systems. Operating aeration on standby and utilizing different aeration units, particularly microaerators and blowers, can be more efficient in BFT technology. Moreover, BFT systems are susceptible to adverse weather conditions like windstorms and hurricanes [117]. To tackle these challenges, alternative energy sources can be employed to decrease the significant electricity consumption involved in intensive aeration and pumping, thereby reducing operating expenses.

The selection of a suitable, cost-effective, readily available, and degradable carbon source is also crucial. Different carbon sources stimulate bacterial activity and have an impact on the microbial composition, community organization, and nutritional properties of bioflocs. Therefore, proper monitoring and selection of carbon sources (as a cost component in biofloc systems) are vital to ensure fish performance and water quality in biofloc ponds [43, 143]. In general, biofloc farming requires high energy demand, initial/implementation costs, effluent and sludge (solids) issues, lack of aquatic/microbial dynamics understanding, skills/qualified labor scarcity, and infrastructure implementation and maintenance costs [22]. Other challenges include reduced response time due to elevated water respiration rates, increased instability of nitrification, and inconsistent and seasonal performance for sunlight-exposed systems [18, 33].

## 5. Future Challenges and Perspectives

BFT has emerged as a promising and sustainable aquaculture technique with significant economic and environmental benefits. However, there are still challenges and opportunities for improvement in BFT systems. One area of future research is the optimization of carbon sources for biofloc production. Specific carbon sources can stimulate particular microbial populations, affecting the nutritional properties of the biofloc and fish performance. Therefore, developing strategies for selecting and managing carbon sources are necessary to improve biofloc quality and quantity. Another critical challenge is managing microbial populations. Imbalances in the microbial community can lead to the accumulation of toxic compounds like nitrate and ammonia, leading to negative impacts on fish health and water quality. Therefore, controlling microbial populations and maintaining a balanced community is essential for the successful implementation of BFT. Effective monitoring tools for BFT systems are also necessary to ensure stable and consistent production. Currently, there are limited techniques available to monitor the microbial community in real-time and detect changes in water quality. Developing accurate and reliable monitoring tools is essential for the successful implementation of BFT. Finally, scaling up BFT systems to commercial production levels is another challenge. Optimizing system design, management practices, and developing economically viable production models are necessary for the successful commercialization of BFT systems.

BFT system perspective can be managed with very high stocking densities and little or no water exchange, which is sustainable in terms of land and water use, with minimal discharges to receiving ecosystems [36]. BFT requires more investment than traditional aquaculture, but the economic analysis shows the technology is feasible. This system provides a quick return on investment due to its high productivity. Furthermore, the risk of contracting diseases is lower, water is used more efficiently, and wastewater is less likely to enter the environment [207]. BFT requires proper management because of its technological and biological complexity. To minimize risks in their overall farming portfolio, farmers can diversify their farming operations with shrimp/fish farming in addition to other existing agricultural enterprises. Methodologies for assessing the environmental impacts of products and production systems could complement aquaculture and agribusiness decision-making processes from an environmental perspective. An investor's decision-making can be informed by methods that compare the enterprise's environmental impact. In order to reduce the environmental impact of a system, we propose the methodology of life cycle assessment, which can be used to identify the critical points and compare different systems to determine which alternative has the least environmental impact [208, 209].

The BFT system is widely recognized for its positive sustainability indicators, which include measures such as FCR, protein efficiency, nitrogen, and phosphorus emissions per ton of protein produced, land use efficiency, and freshwater consumption per ton of production. These indicators play a crucial role in evaluating the environmental impact and efficiency of the biofloc system in aquaculture production.

## 6. Conclusion

BFT has garnered significant attention as an effective solution for meeting the growing global demand for protein. This is due to its year-round production capabilities, location flexibility, and lower environmental impact in comparison to conventional aquaculture practices and wild-caught seafood. BFT systems facilitate zero water exchange, reduce water treatment costs by up to 30%, shorten the cultivation period, and enhance the survival and growth rates of aquatic species, making it a sustainable production system. Moreover, BFT systems have demonstrated economic efficiency by exhibiting lower operational costs, a higher return on investment, and reduced expenses related to water, feed, and the environment, in comparison to conventional culture systems. However, the profitability of a BFT farm may be affected by changes in input factors and biological parameters such as stocking density, production, growth performance, and survival rate. Thus, to succeed, standardizing BFT technology and increasing research in production economics and management are crucial. To sum up, BFT technology presents a viable solution for the ever-increasing demand for protein. Its advantages over conventional aquaculture practices and wild-caught seafood are numerous, including its sustainability, cost-effectiveness, and efficiency. Nevertheless, BFT ventures must continue to focus on standardizing their technology and increasing research in production economics and management to ensure long-term success and growth. The ongoing development and innovation of BFT technology will contribute to the creation of more efficient and sustainable aquaculture practices, benefiting both the industry and the environment.

## Figures and Tables

**Table 1:** A summary of research on the utilization of biofloc in the diets of various reared aquatic species.

Reared species	IW (g)	Biofloc source	Substituted amounts in diet	Suitable amount	Highlights	Authors name and references
*L. vannamei*	2.48	Biofloc meal + soy protein concentrate	0%–100%	100%	Improved growth performance and survival rates	Bauer et al. [96]
*P. monodon*	2.90	Dried biofloc	0%–12%	4%–8%	Improved growth performance and digestive enzyme activities	Anand et al. [98]
*L. vannamei*	7.76	Biofloc meal	0%–15%	15%	Improved growth performance	Shao et al. [87]
*O. niloticus*	2	Biofloc meal	20%–40%	20%	Improved growth performance	Prabu et al. [99]
*P. clarkii*	0.0078	Biofloc meal	33%–100%	33%–66%	Improved growth performance	Lunda et al. [100]
*F. merguiensis*	0.0045	Wet biofloc biomass	25%–100%	25%–50%	Improved growth performance and survival rates	Khanjani and Sharifinia [52]
*P. vannamei*	0.03	biofloc meal	0%–40%	30%	Improves the growth performance and physiological responses of shrimp	Nethaji et al. [101]
*O. niloticus*	21.52	Synbiotic and biofloc meal	3 g/kg feed	—	Improved growth performance and also improve gut microbiota, body composition and tissue histomorphology	Hersi et al. [102]
*L. vannamei*	0.0023	Biofloc meal	7.5%–30%	20%<	Improved growth performance	Dantas et al. [103]
*O. niloticus*	0.0001	Dried bioflocs	0%–16%	4%	Provided higher growth rates and better water quality parameters	Binalshikh-Abubkr, and Hanafiah [104]
*L. vannamei*	5.23	Wet and dried biofloc	5% and 10%	10%	Improved growth performance and FCR	Barzamini et al. [105]
*C. mrigala*	8.33	Microfloc meal	0%, 30%, 40%, and 50%	50%	Improved growth performance, feed utilization, and nonspecific immune response of mrigal	Rani et al. [106]

Abbreviations: IW, initial weight; FCR, feed conversion ratio.

**Table 2:** Growth performance, survival, feed conversion ratio, and production rate have been reported for various species in different cultivation systems in various studies.

Rearing system	Reared aquatic animal	IAW (g)	Rearing duration (days)	Stocking density	Survival (%)	FAW (g)	FCR	Production	Highlight	Authors name and references
Biofloc	Shrimp	—	160	60 shrimp/m^2^	95	39	1.4	19.78 t/ha	Improvement in production was observed in the biofloc system compared to the conventional system	Anand et al. [109]
Clear water	Shrimp	—	149	60 shrimp/m^2^	85	31.68	1.6	15.73 t/ha		
SSF (<1 ha)	Tilapia	—	—	29,100 fish/ha	—	—	1.59	15,040 kg/ha	The results indicated that large farms have the highest cost and the highest cost-profit margin among the three categories, and the small farms have the lowest cost and profit margin, while the cost and profit margin of medium size are between the large and small sizes	Yuan et al. [110]
MSF (1–10 ha)	Tilapia	—	—	37,200 fish/ha	—	—	1.58	18,150 kg/ha		
LSF (>10 ha)	Tilapia	—	—	40,060 fish/ha	—	—	1.51	19,800 kg/ha		
Conventional	*L. vannamei*	—	90	20 shrimp/m^2^	73	10.4	1.4	1,537.0 kg/ha	There was a greater influence of the management inefficiency on the conventional system, whereas the production scale influenced the reduction in technical efficiency score of the BFT system	Rego et al. [111, 112]
Biofloc	*L. vannamei*	—	98	113 shrimp/m^2^	58	11.9	1.8	7,775.0 kg/ha		
Biofloc	*C. chanos*	0.96	30	166.6 fish/m^3^	80	4.70	0.80	—	The study shows that the biofloc-based nursery rearing of milkfish is economically viable and more profitable to the clear-water culture system and can be practiced instead of traditional pond culture	Sontakke and Haridas [24]
Earthen pond	*C. chanos*	0.97	30	166.6 fish/m^3^	55.90	5.20	1.45	—		
Clear water	*C. chanos*	0.98	45	166.6 fish/m^3^	52	4.55	1.2	—		
Conventional	Gibel carp	20.65	60	138.8 fish/m^3^	77.8	40.56	1.9	778 kg/year	BFT system was more effective than the control system with water exchange in gibel carp culture, suggesting that BFT system could be successfully used in gibel carp aquaculture	Cang et al. [113]
Biofloc	Gibel carp	19.09	60	138.8 fish/m^3^	90	44.67	1.6	2,250 kg/year		
Biofloc	Shrimp	—	90	130 shrimp/m^2^	89.16	18.78	1.39	22.51 t/ha	Improvement in production was observed in the biofloc system compared to two other systems	Panigrahi et al. [114]
Semibiofloc	Shrimp	—	101	110 shrimp/m^2^	81.35	18.31	1.58	16.2 t/ha		
Clear water	Shrimp	—	111	83 shrimp/m^2^	83.19	17.80	1.77	12.02 t/ha		
Biofloc	Shrimp	—	—	125–150 shrimp/m^2^	—	—	—	20–25 t/ha	Production increases with higher stocking densities in the biofloc system	Hargreaves [18]
Biofloc	Tilapia	—	—	20–25 fish/m^3^	—	—	—	15–20 (up to 30) kg/m^3^		
Biofloc	*O. niloticus*	30	140	80 fish/m^3^	100	192.9	1.6	15.4 kg/m^3^	Biofloc improved growth performance and protein utilization that led to increase net income	Hwihy et al. [115]
Clear water	*O. niloticus*	30	140	80 fish/m^3^	87.5	168.3	1.9	11.8 kg/m3		
Biofloc	*O. niloticus*	5	60	750 fish/m^3^	80	—	1.13	16.18 kg/m^3^	The tilapia juveniles production in BFT is a low-risk and economically viable investment	Bezerra et al. [116]
Biofloc	*L. vannamei*	—	—	179.11 shrimp/m³	80	12	1.6	5.16 kg/m^3^	Intensive shrimp farming in the biofloc system yields higher net profits	Almeida et al. [117]
Biofloc	*L. vannamei*	—	—	400 shrimp/m³	80	12	1.6	11.52 kg/m^3^		
FLOCponics	*O. niloticus*	—	46	300 fish/m^3^	98	—	0.85	—	The results suggest that it is economically feasible to produce tilapia juveniles and butter lettuce in a conventional aquaponics system	Pinho et al. [57]
Conventional aquaponics	*O. niloticus*	—	46 and 23	300 fish/m^3^ and 20 plants/m^2^	98	—	0.88	—		
Water exchange	*Clarias gariepinus*	7–8	70	300 fish/m^2^	79.4	111.11–142.86	—	168.0 kg/m^2^	The production and financial performance of catfish farming using biofloc technology produces the lowest value compared to the other two systems	Diatin et al. [9]
Aquaponic	*C. gariepinus*	7–8	70	300 fish/m^2^	80	111.11–142.86	—	167.0 kg/m^2^		
Biofloc	*C. gariepinus*	7–8	90	300 fish/m^2^	74.7	111.11–142.86	—	125.0 kg/m^2^		
Biofloc	*L. vannamei*	1.2	75	42 shrimp/m^2^	64.61	11.14	1.42	2,979 kg/ha	The profitability was higher in the (150-day long cycle) treatment due to the higher sale price achieved by the larger size of the shrimp	Poersch et al. [79]
Biofloc	*L. vannamei*	1.2	150	42 shrimp/m^2^	68.01	26.41	1.57	7,543 kg/ha		
Clear water	*L. vannamei*	2.56	35	1 kg/m^3^	83.2	6.82	1.6	1.4 kg/m^3^	Farming in the biofloc system leads to improvement in production, survival, and FCR	Khanjani et al. [118]
Biofloc	*L. vannamei*	2.56	35	1 kg/m^3^	90.2	7.25	0.9	1.7 kg/m^3^		
Biofloc	*O. niloticus*	1.79	35	1.79 kg/m^3^	98.82	7.94	1	6.1 kg/m^3^	Improvement in survival and FCR was observed in the biofloc system compared to the conventional system	Khanjani and Alizadeh [119]
Clear water	*O. niloticus*	1.79	35	1.79 kg/m^3^	96.86	7.47	1.54	5.5 kg/m^3^		
Biofloc	*L. vannamei*	—	360	400 shrimp/m^2^	80	12	—	69,120 kg/year (three cycles/year)	The analysis confirms that *L. vannamei* production using the BFT system is a financially sustainable activity	Almeida et al. [120]
Clear water	*L. vannamei*	3.5	30	312 juveniles/m^3^	92.13	7.37	1.52	2.12 kg/m^3^	The results of this study suggest that culture in biofloc-enriched water produces higher levels of water quality and shrimp performance than culture in natural seawater	Krummenauer et al. [20]
Biofloc					90.93–99.06	8.01–8.42	0.84–1.23	2.35–2.48 kg/m^3^		
Reused water biofloc	*O. niloticus*	78.74	98	100 fish/m^3^	98.75	163.09	2.22	16.30 kg/m^3^	The results suggest that the intensive cultivation of Nile tilapia in biofloc can be established using reuse water from BFT systems, without adverse effects on their survival, productive performance, proximal composition, and gonadal development	Gallardo-Collı et al. [121]
Biofloc		79.83	98		100	159.23	2.40	15.63 kg/m^3^		
Biofloc	*O. niloticus*	1.17	42	500 fish/m^3^	100	18.99	—	9.49 kg/m^3^	It is possible to increase the stocking density of Nile tilapia fingerlings up to 1000 fish/m³ in a low salinity biofloc system, since this has no effect on the zootechnical potential. Also with a positive effect of stocking density increase on yield	Lima et al. [19]
				750 fish/m^3^	100	17.47	—	13.28 kg/m^3^		
				1,000 fish/m^3^	99.33	15.36	—	15.27 kg/m^3^		
				1,250 fish/m^3^	96.82	12.40	—	15.12 kg/m^3^		
Biofloc	Rohu, *L. rohita*	53.33	90	1.3 fish/m^2^	100	140	1.93	5.6 kg/700 L	This study demonstrates the potential of biofloc technology in the culture of *rohu, L. rohita*. The highest net yield obtained in the biofloc treatment was almost 87% more than that in corresponding control treatment	Mahanand et al. [122]
		52.5		2.6 fish/m^2^		103.95	3.55	8.3 kg/700 L		
		48.61		3.9 fish/m^2^		97.36	3.66	11.7 kg/700 L		
Clear water		56.75		1.3 fish/m^2^		112.75	2.54	4.5 kg/700 L		
		58.41		2.6 fish/m^2^		93.29	3.76	7.5 kg/700 L		
		54.86		3.9 fish/m^2^		80.86	4.58	9.7 kg/700 L		
Clear water	*L. rohita*	26.93	90	4.28 fish/m^3^	—	49.21	2.78	—	The implementation of the BFT at a stocking density of 12.85 fish/m^3^ resulted in excellent water quality management with a reduced number of water exchanges, feed recycling, and improved growth of the cultured species when compared to the control treatment. Furthermore, FCR values in the BFT system at all stocking densities were significantly lower than those in the control system, indicating a higher efficiency in feed utilization	Deb et al. [85]
		27.07		12.85 fish/m^3^	—	46.87	3.15	—		
Biofloc		26.56		4.28 fish/m^3^	—	64.16	1.69	—		
		26.95		12.85 fish/m^3^	—	50.14	2.12	—		
Clear water	*C.catla*	20.46		4.28 fish/m^3^	—	36.49	2.92	—		
		19.8		12.85 fish/m^3^	—	33.47	3.25	—		
Biofloc		19.2		4.28 fish/m^3^	—	51.70	1.61	—		
		13.58		12.85 fish/m^3^	—	32.03	2.01	—		
Clear water	*C. mrigala*	30.53		4.28 fish/m^3^	—	52.03	2.56	—		
		26.93		12.85 fish/m^3^	—	50.02	2.77	—		
Biofloc		28.76		4.28 fish/m^3^	—	59.71	2.23	—		
		29.06		12.85 fish/m^3^	—	52.73	2.76	—		
Clear water	*L. vannamei*	0.18	119	350 individuals/m^2^	93.84	10.58	1.49	3.08 kg/m^3^	The survival, productivity, and FCR were significantly better in the BFT treatments compared to those of the control	Gaona et al. [84]
Biofloc					98	11.51–11.65	1.23–1.25	4.5 kg/m^3^		
Biofloc	*L. vannamei*	1.78	28	300 shrimps/m^3^	—	7.37–11.01	1.17–1.80	1.69–2.65 kg/m^3^	The results indicate that it is possible to submit *L. vannamei* to partial feed restriction with a later recovery period as a trigger for total compensatory growth in BFT system	Prates et al. [123]
Conventional system (CS)	*O. niloticus*	16.67	98	120 fish/m^3^	—	40.00	1.89	—	BFT improved the Nile tilapia fingerlings' growth compared to the CS. Applying BFT and probiotic could improve the efficiency of tilapia culture. Symbiosis of BFT-10 : 1 with *B.subtilis* probiotics accomplished the highest growth performance parameters with a pronounced improvement in feed utilization	Haraz et al. [86]
CS + *B. subtilus*		16.73			—	42.27	1.80	—		
BFT, C/N = 10		16.48			—	50.67	1.54	—		
BFT, C/N = 10 + *B. subtilus*		16.53			—	57.33	1.41	—		
BFT, C/N = 20		16.82			—	45.72	1.62	—		
BFT, C/N = 20 + *B. subtilus*		16.76			—	47.38	1.56	—		
BFT, C/N = 8	*L. vannamei*	0.79	63	270 individuals/m^3^	81.5	12.46	1.24	2.73	The results suggested that the C : N higher than 16 were suitable for culturing *L. vannamei* in the biofloc system with a salinity of 5‰, with an optimal range of 18.5–21.0 : 1	Huang et al. [124]
BFT, C/N = 16		0.83			96.8	11.71	1.21	3.06		
BFT, C/N = 24		0.82			93.7	11.27	1.34	2.82		

Abbreviations: FAW, final average weight; IAW, initial average weight; FCR, feed conversion ratio; CS, conventional system; gibel carp = *Carassius auratus gibelio* ♀ × *C. carpio* ♂; SSF, small scale farming; MSF, medium scale farming; LSF, large scale farming.

**Table 3:** Water consumption in different aquaculture systems.

Rearing system	Reared aquatic animal	IAW (g)	RP (days)	Water consumption	Highlight	Authors name and references
Biofloc	*L. vannamei*	0.085 mg	7	6.49–6.89 L/thousand PL	Treatment with dextrose or molasses required approximately 12% of the water used by the CW group	de Lorenzo et al. [47]
Clear water	—	—	—	56.22 L/thousand PL		
Conventional	Gibel carp	20.65	60	31.7 m^3^/kg	Water usage in the biofloc system is significantly lower compared to the conventional system	Cang et al. [113]
Biofloc	Gibel carp	19.09	60	0.30 m^3^/kg		
Biofloc	*O. niloticus*	30	140	108 L/kg	BFT provides 85% of water consumption vs. control clear water groups, so it is a comprehensive promising technology suitable for aquaculture away from water bodies	Hwihy et al. [115]
Clear water	*O. niloticus*	30	140	1,166 L/kg		
FLOCponics	*O. niloticus*	—	46	2%	Water usage in the aquaponic system is negligible	Pinho et al. [57]
Conventional aquaponics	*O. niloticus*	—	46 and 23	1.9%		
Clear water	*Clarias gariepinus*	7–8 g	70	As much as 20% every morning and evening	The water consumption in the aquaponic system was negligible, and the water was circulated. Water usage in the biofloc system is lower compared to two other systems	Diatin et al. [9]
Aquaponic	*C. gariepinus*	7–8 g	70	Water circulation: 1,500 L/hr		
Biofloc	*C. gariepinus*	7–8 g	90	Zero		
Biofloc	*L. vannamei*	1.2	75	No water renewal occurred, only replacement of water lost by evaporation	Water usage in the biofloc system is negligible and only replaced with evaporated water	Poersch et al. [79]
Biofloc	*L. vannamei*	1.2	150			
Clear water	*L. vannamei*	2.56	35	Daily 35%–50% of the volume of rearing water	Water usage in the biofloc system is 35%–50% less per day than in the conventional system	Khanjani et al. [118]
Biofloc	*L. vannamei*	2.56	35	Zero		
Biofloc	*O. niloticus*	1.79	35	61.47 L/kg	Water usage in the biofloc system was 40% less per day than in the conventional system	Khanjani and Alizadeh [119]
Clear water	*O. niloticus*	1.79	35	2777.64 L/kg		
Biofloc	*O. niloticus*	1.17	42	52.48–101.54 L/kg	In the BFT, the amount of water consumption increases with the increase in stocking density	Lima et al. [19]
Biofloc	Indian major carp	Rohu = 26.33 Catla = 17.3 Mrigal = 28.1	90	1,050–1,400 L/TWR	Water requirement in BFT was ∼37.8% less as compared to CW	Deb et al. [85]
Clear water				1,750–2,100 L/TWR		
Biofloc	Rohu, *L. rohita*	50	90	133.7–208.5 L/kg	The needed number of water exchanges was more in treatments with higher stocking densities and less in biofloc treatments. The amount of water consumption in the BFT (according to the stoking density) was 34%–40% less than the control group	Mahanand et al. [122]
Clear water				200.5–345 L/kg		
Water renewal	*L. vannamei*	0.83	30	2.22 m^3^/kg	The use of clarifiers helps to control TSS concentrations in large scale. They reduce both the amount of water used for renewals and the effluent discharges into the environment, thereby increasing biosafety in the BFT	Zemor et al. [140]
BFT, with clarifier	—	—	—	1.18–1.2 m^3^/kg		
Biofloc	*O. niloticus*	55.2	49	Different rates of water reuse (0%, 25%, 50%, 75%, and 100%)	The results suggest that it is possible to reuse biofloc water, positively affecting water quality and productive response, and causing no adverse effect on the fish's health	Figueroa-Espinoza et al. [21]

Abbreviations: IAW, initial average weight; WC, water consumption; RP, rearing period; CW, clear water; gibel carp = *Carassius auratus gibelio* ♀ × *C. carpio* ♂; PL, post-larvae; indian major car*p* = rohu (*Labeo rohita*), catla (*Catla catla*), mrigal (*Cirrihinus mrigala*); TWR. total water requirement.

**Table 4:** Some of the studies conducted in line with the economic goals of the biofloc system, costs are considered in terms of total production costs.

Reared aquatic animal	Rearing duration (days)	IAW (g)	Stocking density	Implementation (US$/m^2^ or m³)	Labor (%/TC)	Electrical energy (%/TC)	Commercial feed (%/TC)	larvae (%/TC)	Carbon source (%/TC)	Authors name and references
Shrimp	—	—	—	7.56	17.16	—	62.00	15.00	—	Teixeira and Guerrelhas [160]
Shrimp	—	—	120–180 shrimp/m^2^	8.79	3.68	7.45	62.22	13.71	—	Poersch et al. [151]
Tilapia	—	—	29,100–40,060 fish/ha	—	5.98	2.52	66.11	4.58	—	Yuan et al. [110]
*L. vannamei*	—	—	113 shrimp/m^2^	14.83	13.66	14.46	37.89	17.63	—	Rego et al. [111, 112]
Gibel carp	60	19.09	138.8 fish/m^3^	—	0.86	13	65.00	10.82	6.5	Cang et al. [113]
*L. vannamei*	56	0.01	400 shrimp/m^2^	16.23	21.52	6.39	53.17	14.71	—	Mauladani et al. [157]
*L. vannamei*	—	—	179.11 shrimp/m³	18.83	14.39	11.80	59.73	8.83	1.59	Almeida et al. [117]
*L. vannamei*	—	—	400 shrimp/m³	48.76	14.87	9.34	61.74	9.13	0.74	Almeida et al. [117]
*O. niloticus*	140	30	80 fish/m^3^	46.31	—	—	82.4	9.73	—	Hwihy et al. [115]
*Chanos chanos*	30	0.96	166.6 fish/m^3^	—	4.43	—	6.4	46.13	3.72	Sontakke and Haridas [24]
*O. niloticus*	60	5	750 fish/m^3^	17.2	7.09	1.49	22.42	33.24	0.38	Bezerra et al. [116]
*C. gariepinus*	90	7–8	300 fish/m^2^	—	39.50	1.97	31.60	22.11	1.05	Diatin et al. [9]
*L. vannamei*	75	1.2	42 shrimp/m^2^	—	—	13	69	18	—	Poersch et al. [79]
*L. vannamei*	150	1.2	42 shrimp/m^2^	—	—	12	80	0.08	—	Poersch et al. [79]
*L. vannamei*	36	—	1,000 shrimp/m^2^	—	3	15	22	47	3	Tailly [161]
*O. niloticus*	35	1.79	1.79 kg/m^3^	26.5	—	—	40.36	26.6	6.6	Khanjani and Alizadeh [119]
*L. vannamei*	35	2.56	1 kg/m^3^	23.8	—	—	53.13	17.46	5.5	Khanjani et al. [118]
*L. vannamei*	—	—	400 shrimp/m^2^	—	17.06	10.70	57.54	10.47	—	Almeida et al. [120]

Abbreviations: IAW, initial average weight; TC, total costs; gibel carp = *Carassius auratus gibelio* ♀ × *C. carpio* ♂.

**Table 5:** A compilation of various studies that have investigated the levels of total suspended solids in the biofloc system.

Reared species	IAW (g)	RP (days)	Carbon source used	C/N ratio	Amount of TSS (mg/L)	Highlight	Authors name and references
*Rhamdia quelen*	—	21	Dextrose	20 : 1	200	The findings indicate that *R. quelen* larvae can successfully be grown in a BFT system with TSS concentrations of up to 1,000 mg/L	Poli et al. [189]
					400–600		
					800–1,000		

*Piaractus mesopotamicus*	20.70	4	Cane molasses	15 : 1	0	The study demonstrated that TSS concentrations below 5,000 mg/L did not result in any mortality, whereas a TSS concentration of 6,000 mg/L caused 25% mortality, and all fish exposed to a TSS concentration of 7,000 mg/L died. These findings suggest that pacu juveniles can tolerate TSS concentrations of up to 5,000 mg/L, which is higher than what is typically used in BFT systems. This resistance to higher TSS concentrations may be advantageous for pacu farmers who use BFT systems, as it provides a wider range of flexibility in managing water quality. However, caution must still be exercised to ensure that TSS concentrations do not reach levels that may compromise the health and growth of the fish. Further research is needed to determine the optimal TSS concentration range for pacu juveniles in BFT systems	Pellegrin et al. [190]
					1,500		
					3,000		
					4,000		
					5,000		
					6,000		
					7,000		

*Piaractus mesopotamicus*	28.41	5	Cane molasses	15 : 1	0	Exposure of pacu to high TSS concentrations for short periods may induce physiological changes, which can negatively impact fish health and growth. Therefore, it is recommended that TSS concentrations below 250 mg/L should be maintained in BFT systems for pacu production. While pacu juveniles have been shown to exhibit some tolerance to higher TSS concentrations, a prolonged exposure to such conditions may cause stress and lead to reduced growth rates, decreased immune function, and increased susceptibility to diseases. Therefore, it is essential to manage TSS concentrations carefully in BFT systems to ensure optimal fish health and growth	Pellegrin et al. [190]
					250		
					500		
					750		

*L. vannamei*	0.18	119	Molasses from sugarcane	6 : 1	500–600	The study found that the use of low water flow through the clarifier resulted in better removal efficiency, as it allowed for a higher retention rate of particles in the settling chamber. This method can be effective in reducing the amount of suspended solids in the water and improving water quality in aquaculture systems, particularly for shrimp production. Furthermore, the study suggested that the practice of not changing the water in culturing tanks after water withdrawal can optimize the use of hydric resources and improve the overall efficiency of shrimp production systems. This practice reduces the amount of water needed for production, which can lead to significant cost savings and a more sustainable use of resources	Gaona et al. [84]

*L. vannamei*	4.54	42	Sugar cane molasses	20 : 1	100–300	The study showed that maintaining a lower range of TSS concentrations in the BFT system resulted in better performance of *L. vannamei* shrimp. Specifically, TSS concentrations in the range of 100–300 mg/L were found to be optimum for shrimp culture. This finding suggests that maintaining appropriate TSS concentrations is critical for the success of shrimp production in BFT systems. High TSS concentrations can negatively impact the health and growth of shrimp by reducing dissolved oxygen levels, increasing ammonia concentrations, and promoting the growth of harmful bacteria	Gaona et al. [186]
					300–600		
					600–1,000		

*Penaeus vannamei*	—	14	—	—	300	The study found that maintaining a TSS level of less than 300 mg/L in the BFT system can lead to better performance of *P. vannamei* nauplius in the nursery stage. This result suggests that maintaining appropriate TSS concentrations is critical for the successful cultivation of *P. vannamei* nauplius in BFT systems. High TSS concentrations can negatively impact the health and growth of shrimp by reducing dissolved oxygen levels, increasing ammonia concentrations, and promoting the growth of harmful bacteria	Liu et al. [191]
			—	—	600		

*Carassius auratus* gibelio	6.4	30	Molasses	15 : 1	10	The study demonstrated that bioflocs in the culture system contained rich nutrients, and feeding gibel carp with bioflocs resulted in higher weight gain, specific growth, and survival rates compared to the control group. Moreover, digestive enzyme activities, such as pepsin and amylase, were significantly increased in the BF300/600/800/1000-NF groups compared to the control group. In addition to improved digestive function, the study found that the biofloc-fed gibel carp exhibited enhanced antioxidant responses, including significantly increased levels of superoxide dismutase and total antioxidant capacity in both serum and skin mucus. This suggests that feeding gibel carp with bioflocs may enhance their overall health and immunity	Zhang et al. [76]
					300		
					600		
					800		
					1,000		

*L. vannamei*	6.2	35	Molasses	6 : 1	30	The study found that the cultivation of *Ulva lactuca* in shrimp effluent water with a TSS concentration of 30 mg/L resulted in significantly lower TSS concentrations compared to the BFT groups, which had a TSS concentration of 400 mg/L. However, turbidity showed similar results between the treatment with 30 mg/L of TSS and the BFT groups. These findings suggest that the cultivation of *U. lactuca* can be an effective method for reducing TSS concentrations in shrimp effluent water. This method can be used to improve water quality and reduce environmental impacts associated with shrimp farming. While TSS concentrations were significantly reduced in the treatment group, turbidity levels were not significantly different from those observed in the BFT groups. This may be due to the presence of other suspended particles, such as organic matter and microbial biomass, in the BFT groups	Carvalho et al. [192]
					400		

Abbreviations: IW, initial weight; RP, rearing period; TSS, total suspended solids.

## Data Availability

Data supporting this review article are available on request.

## References

[1] Valenti W. C., Kimpara J. M., Preto B. L., Moraes-Valenti P. (2018). Indicators of sustainability to assess aquaculture systems. *Ecological Indicators*.

[2] Custodio M., Villasante S., Calado R., Lillebø A. I. (2020). Valuation of ecosystem services to promote sustainable aquaculture practices. *Reviews in Aquaculture*.

[3] Filipski M., Belton B. (2018). Give a man a fishpond: modeling the impacts of aquaculture in the rural economy. *World Development*.

[4] Sharifinia M., Bahmanbeigloo Z. A., Keshavarzifard M. (2023). The effects of replacing fishmeal by mealworm (*Tenebrio molitor*) on digestive enzymes activity and hepatopancreatic biochemical indices of *Litopenaeus vannamei*. *Annals of Animal Science*.

[5] Sharifinia M., Bahmanbeigloo Z. A., Keshavarzifard M. (2023). Fishmeal replacement by mealworm (*Tenebrio molitor*) in diet of farmed Pacific white shrimp (*Litopenaeus vannamei*): effects on growth performance, serum biochemistry, and immune response. *Aquatic Living Resources*.

[6] Da Silva K. R., Wasielesky W., Abreu P. (2013). Nitrogen and phosphorus dynamics in the biofloc production of the Pacific white shrimp, *Litopenaeus vannamei*. *Journal of the World Aquaculture Society*.

[7] Weidner D., Rosenberry B., Wyban J. (1992). *World Shrimp Farming: Proceeding of the Special Session on Shrimp Farming*.

[8] Lorenzen K. (1999). Nitrogen recovery from shrimp pond effluent: dissolved nitrogen removal has greater overall potential than particulate nitrogen removal, but requires higher rates of water exchange than presently used. *Aquaculture Research*.

[9] Diatin I., Shafruddin D., Hude N., Sholihah M., Mutsmir I. (2021). Production performance and financial feasibility analysis of farming catfish (*Clarias gariepinus*) utilizing water exchange system, aquaponic, and biofloc technology. *Journal of the Saudi Society of Agricultural Sciences*.

[10] Diatin I., Suprayudi M. A., Budiardi T., Surawidjaja E. H., Widanarni W. (2015). Intensive culture of corydoras ornamental fish (*Corydoras aeneus*): evaluation of stocking density and water exchange. *AACL Bioflux*.

[11] Khanjani M. H., Zahedi S., Mohammadi A. (2022). Integrated multitrophic aquaculture (IMTA) as an environmentally friendly system for sustainable aquaculture: functionality, species, and application of biofloc technology (BFT). *Environmental Science and Pollution Research*.

[12] Tórz A., Burda M., Półgęsek M., Sadowski J., Nędzarek A. (2022). Transformation of phosphorus in an experimental integrated multitrophic aquaculture system using the media filled beds method in plant cultivation. *Aquaculture Environmental Interaction*.

[13] Sharifinia M., Penchah M. M., Mahmoudifard A., Gheibi A., Zare R. (2015). Monthly variability of chlorophyll-*α* concentration in Persian gulf using remote sensing techniques. *Sains Malaysiana*.

[14] Debbarma R., Meena D. K., Biswas P., Meitei M. M., Singh S. K. (2022). Portioning of microbial waste into fish nutrition via frugal biofloc production: a sustainable paradigm for greening of environment. *Journal of Cleaner Production*.

[15] Minaz M., Yazıcı İ. S., Sevgili H., Aydın İ. (2023). Biofloc technology in aquaculture: advantages and disadvantages from social and applicability perspectives. *Annals of Animal Science*.

[16] Khanjani M. H., Sharifinia M., Hajirezaee S. (2023). Biofloc: a sustainable alternative for improving the production of farmed cyprinid species. *Aquaculture Reports*.

[17] Avnimelech Y. (2012). *Biofloc Technology—A Practice Guide Book*.

[18] Hargreaves J. A. (2013). Biofloc production systems for aquaculture. *Southern Regional Aquaculture Center*.

[19] Lima P. C. M., Abreu J. L., Silva A. E. M., Severi W., Galvez A. O., Brito L. O. (2018). Nile tilapia fingerling cultivated in a low-salinity biofloc system at different stocking densities. *Spanish Journal of Agricultural Research*.

[20] Krummenauer D., Samocha T., Poersch L., Lara G., Wasielesky W. (2014). The reuse of water on the culture of pacific white shrimp, *Penaeus vannamei*, in BFT system. *Journal of the World Aquaculture Society*.

[21] Figueroa-Espinoza J., Rivas-Vega M. E., Mariscal-López M. A., Emerenciano M. G. C., Martínez-Porchas M., Miranda-Baeza A. (2022). Reusing water in a biofloc culture system favors the productive performance of the Nile tilapia (*Oreochromis niloticus*) without affecting the health status. *Aquaculture*.

[22] Khanjani M. H., da Silva L. O. B., Foes G. K. Synbiotics and aquamimicry as alternative microbial-based approaches in intensive shrimp farming and biofloc: novel disruptive techniques or complementary management tools? A scientific-based overview. *Aquaculture*.

[23] Andrade T., Afonso A., Perez-Jimenez A. (2015). Evaluation of different stocking densities in a Senegalese sole (*Solea senegalensis*) farm: implication for growth, humoral immune parameters and oxidative status. *Aquaculture*.

[24] Sontakke R., Haridas H. (2018). Economic viability of biofloc based system for the nursery rearing of milkfish (*Chanos chanos*). *International Journal of Current Microbiology and Applied Sciences*.

[25] Megahed M. E. (2010). The effect of microbial biofloc on water quality, survival and growth of the green tiger shrimp (*Penaeus semisulcatus*) fed with different crude protein levels. *Journal of the Arabian Aquaculture Society*.

[26] De Schryver P., Verstraete W. (2009). Nitrogen removal from aquaculture pond water by heterotrophic nitrogen assimilation in lab-scale sequencing batch reactors. *Bioresource Technology*.

[27] Emerenciano M. G. C., Arnold S., Perrin T. (2022). Sodium metasilicate supplementation in culture water on growth performance, water quality and economics of indoor commercial-scale biofloc-based *Litopenaeus vannamei* culture. *Aquaculture*.

[28] Walker D. A. U., Morales-Suazo M. C., Emerenciano M. G. C. (2020). Biofloc technology: principles focused on potential species and the case study of Chilean river shrimp *Cryphiops caementarius*. *Reviews in Aquaculture*.

[29] Emerenciano M. G. C., Arnold S., Perrin T., Little B., Cowley J. A., Rahman A. (2022). Collaboration drives innovations in super-intensive indoor shrimp farming, part 2. https://www.globalseafood.org/advocate/collaboration-drives-innovations-in-super-intensive-indoor-shrimp-farming-part-2/.

[30] Khanjani M. H., Mozanzade M. T., Sharifinia M., Emerenciano M. G. C. (2023). Biofloc: a sustainable dietary supplement, nutritional value and functional properties. *Aquaculture*.

[31] Khanjani M. H., Mohammadi A., Emerenciano M. G. C. (2022). Microorganisms in biofloc aquaculture system. *Aquaculture Reports*.

[32] Waite R., Beveridge M., Brummett R. (2014). Improving productivity and environmental performance of aquaculture: creating a sustainable food future installment five. http://www.wri.org/publication/improving-aquaculture.

[33] Crab R., Defoirdt T., Bossier P., Verstraete W. (2012). Biofloc technology in aquaculture: beneficial effects and future challenges. *Aquaculture*.

[34] Khanjani M. H., Eslami J., Ghaedi G., Sourinejad I. (2022a). The effects of different stocking densities on nursery performance of banana shrimp (*Fenneropenaeus merguiensis*) reared under biofloc condition. *Annals of Animal Sciences*.

[35] Khanjani M. H., Mozanzade M. Torfi, Fóes G. K. (2022c). Aquamimicry system: a suitable strategy for shrimp aquaculture. *Annals of Animal Scienses*.

[36] Martínez-Córdova L. R., Martínez-Porchas M., Emerenciano M. G. C., Miranda-Baeza A., Gollas-Galván T. (2017). From microbes to fish the next revolution in food production. *Critical Reviews in Biotechnology*.

[37] Ogello E. O., Musa S. M., Aura C. M., Abwao J. O., Munguti J. M. (2014). An appraisal of the feasibility of tilapia production in ponds using biofloc technology: a review. *International Journal of Aquatic Science*.

[38] Pérez-Rostro C. I., Pérez-Fuentes J. A., Hernández-Vergara M. P., Hernandez-Vergara M. P., Perez-Rostro C. I. (2014). Biofloc, a technical alternative for culturing Malaysian prawn *Macrobrachium rosenbergii*. *Sustainable Aquaculture Techniques*.

[39] Wasielesky W., Atwood H., Stokes A., Browdy C. L. (2006). Effect of natural production in a zero exchange suspended microbial floc based super-intensive culture system for white shrimp *Penaeus vannamei*. *Aquaculture*.

[40] Taw N. (2010). Biofloc technology expanding at white shrimp farms: biofloc systems deliver high productivity with sustainability. http://www.gaalliance.org/mag/May_June2010.pdf.

[41] Emerenciano M., Gaxiola G., Cuzon G. (2013). Biofloc technology (BFT): a review for aquaculture application and animal food industry. *Biomass Now-Cultivation and Utilization*.

[42] Vázquez-Euán R., Garibay-Valdez E., Martínez-Porchas M. (2022). Effect of different probiotic diets on microbial gut characterization and gene expression of *Litopenaeus vannamei* cultivated in BFT system. *Turkish Journal of Fisheries and Aquatic Sciences*.

[43] Khanjani M. H., Alizadeh M., Mohammadi M., Sarsangi H. Aliabad (2021). Biofloc system applied to Nile tilapia (*Oreochromis niloticus*) farming using different carbon sources: growth performance, carcass analysis, digestive and hepatic enzyme activity. *Iranian Journal of Fisheries Sciences*.

[44] Emerenciano M. G. C., Miranda-Baeza A., Martínez-Porchas M., Poli M. A., Vieira F. N. (2021). Biofloc technology (BFT) in shrimp farming: past and present shaping the future. *Frontiers in Marine Science*.

[45] Khanjani M. H., Sharifinia M. (2020). Biofloc technology as a promising tool to improve aquaculture production. *Reviews in Aquaculture*.

[46] Ferreira G. S., Santos D., Schmachtl F. (2021). Heterotrophic, chemoautotrophic and mature approaches in biofloc system for Pacific white shrimp. *Aquaculture*.

[47] de Lorenzo M. A., Schveitzer R., Santo C. M. (2015). Intensive hatchery performance of the Pacific white shrimp in biofloc system. *Aquacultural Engineering*.

[48] Ekasari J., Rivandi D. R., Surawidjaja E. H., Zairin Jr. M., Bossier P., Schryver P. De (2015). Biofloc technology positively affects Nile tilapia (*Oreochromisniloticus*) larvae performance. *Aquaculture*.

[49] Khanjani M. H., Alizadeh M. (2023). Effects of different salinity levels on performance of Nile tilapia fingerlings in a biofloc culture system. *Annals of Animal Science*.

[50] Garcés S., Lara G. (2023). Applying biofloc technology in the culture of *Mugil cephalus* in subtropical conditions: effects on water quality and growth parameters. *Fishes*.

[51] Promthale P., Pongtippatee P., Withyachumnarnkul B., Wongpraserta K. (2019). Bioflocs substituted fishmeal feed stimulates immune response and protects shrimp from *Vibrio parahaemolyticus* infection. *Fish & Shellfish Immunology*.

[52] Khanjani M. H., Sharifinia M. (2022). Biofloc as a food source for banana shrimp *Fenneropenaeus merguiensis* postlarvae. *North American Journal of Aquaculture*.

[53] Amriawati E., Budiardi T., Setiawati M., Rohmana D., Ekasari J. (2021). Digestive system and growth performance of giant gourami (*Osphronemus goramy* Lacepede) juveniles in biofloc systems fed with different feed types. *Aquaculture Research*.

[54] Ahmed I., Leya T., Saharan N. (2019). Carbon sources affects water quality and haemato-biochemical responses of *Labeo rohita* in zero water exchange biofloc system. *Aquaculture Research*.

[55] Fischer H., Romano N., Renukdas N., Egnew N., Sinha A. K., Ray A. J. (2020). The potential of rearing juveniles of bluegill, *Lepomis macrochirus*, in a biofloc system. *Aquaculture Reports*.

[56] Sudirman A., Rahardjo S., Rukmono D. (2020). Economical analysis of polyculture of catfish and tilapia fish in biofloc system. *The International Journal of Engineering and Science*.

[57] Pinho S. M., Flores R. M. V., David L. H., Emerenciano M. G. C., Quagrainie K. K., Portella M. C. (2022). Economic comparison between conventional aquaponics and FLOCponics systems. *Aquaculture*.

[58] Ray A., Avnimelech Y. (2012). Biofloc technology for super-intensive shrimp culture. *The World Aquaculture Society*.

[59] Miller M. B., Bassler B. L. (2001). Quorum sensing in bacteria. *Annual Review of Microbiology*.

[60] Lazazzera B. A. (2000). Quorum sensing and starvation: signals for entry into stationary phase. *Current Opinion in Microbiology*.

[61] Defoirdt T., Boon N., Bossier P., Verstraete W. (2004). Disruption of bacterial quorum sensing: an unexplored strategy to fight infections in aquaculture. *Aquaculture*.

[62] Crab R., Lambert A., Defoirdt T., Bossier P., Verstraete W. (2010). Bioflocs protect gnotobiotic brine shrimp (*Artemia franciscana*) from pathogenic *Vibrio harveyi*. *Journal of Applied Microbiology*.

[63] Defoirdt T., Sorgeloos P., Bossier P. (2011). Alternatives to antibiotics for the control of bacterial disease in aquaculture. *Current Opinion in Microbiology*.

[64] De Schryver P. (2010). Poly-*β*-hydroxy butyrate as a microbial agent in aquaculture.

[65] Defoirdt T., Halet D., Vervaeren H. (2007). The bacterial storage compound poly-beta-hydroxybutyrate protects *Artemia franciscana* from pathogenic *Vibrio campbellii*. *Environmental Microbiology*.

[66] Ferreira G. S., Bolívar N. C., Pereira S. A. (2015). Microbial biofloc as source of probiotic bacteria for the culture of *Litopenaeus vannamei*. *Aquaculture*.

[67] Maya S. G., Monroy D. M. C., Partida A. H., Mejía J. C., de Oca G. A. R. M. (2016). Effect of two carbon sources in microbial abundance in a biofloc culture system with *Oreochromis niloticus* (Linnaeus, 1758). *International Journal of Fisheries and Aquatic Studies*.

[68] Menaga M., Felix S., Charulatha M., Gopalakannan A., Mohanasundari C., Satyanarayana B. (2020). *In vivo* efficiency of *Bacillus* sp. isolated from biofloc system on growth, haematological, immunological and antioxidant status of genetically improved farmed tilapia (GIFT). *Indian Journal of Exprimental Biology*.

[69] Gustilatov M., Widanarni J. E., Pande G. S. J. (2022). Protective effects of the biofloc system in Pacific white shrimp (*Penaeus vannamei*) culture against pathogenic *Vibrio parahaemolyticus* infection. *Fish & Shellfish Immunology*.

[70] Li Q., Austin B., Sharifuzzaman S. (2022). Probiotics for biofloc system and water quality. *Probiotics in Aquaculture*.

[71] Verschuere L., Rombaut G., Sorgeloos P., Verstraete W. (2000). Probiotic bacteria as biological control agents in aquaculture. *Microbiology and Molecular Biology Reviews*.

[72] Nayak S. K. (2010). Probiotics and immunity: a fish perspective. *Fish & Shellfish Immunology*.

[73] Vieira J. L., Nunes L. S., de Menezes F. G. R., de Mendonça K. V., de Sousa O. V. (2021). An integrated approach to analyzing the effect of biofloc and probiotic technologies on sustainability and food safety in shrimp farming systems. *Journal of Cleaner Production*.

[74] Van Doan H., Lumsangkul C., Ruangwong O. U. (2021). Effects of host-associated probiotic *Bacillus altitudinis* B61-34b on growth performance, immune response and disease resistance of Nile tilapia (*Oreochromis niloticus*) raised under biofloc system. *Aquaculture Nutrition*.

[75] Mohammadi G., Rafiee G., Tavabe K. R., Abdel-Latif H. M., Dawood M. A. (2021). The enrichment of diet with beneficial bacteria (single-or multi-strain) in biofloc system enhanced the water quality, growth performance, immune responses, and disease resistance of Nile tilapia (*Oreochromis niloticus*). *Aquaculture*.

[76] Zhang M., Li Y., Xu D. H. (2018). Effect of different water biofloc contents on the growth and immune response of gibel carp cultured in zero water exchange and no feed addition system. *Aquaculture Research*.

[77] Ju Z., Forster I., Conquest L., Dominy W. (2008). Enhanced growth effects on shrimp (*Litopenaeus vannamei*) from inclusion of whole shrimp floc or floc fractions to a formulated diet. *Aquaculture Nutrition*.

[78] Xu W. J., Pan L. Q. (2013). Enhancement of immune response and antioxidant status of *Litopenaeus vannamei* juvenile in biofloc-based culture tanks manipulating high C/N ratio of feed input. *Aquaculture*.

[79] Poersch L. H., Magalhães V., Lara G., Chaves F., Wasielesky W., Fóes G. K. (2021). Comparative strategies for intensive shrimp production in ponds using biofloc technology system in Southern Brazil: water quality, zootechnical performance and economic viability for *Litopenaeus vannamei*. *Aquaculture Research*.

[80] Emerenciano M. G. C., Martinez-Cordova L. R., Martinez-Porchas M., Miranda-Baeza A., Tutu H., Grover B. P. (2017). Biofloc technology (BFT): a tool for water quality management in aquaculture. *Water Quality*.

[81] Ekasari J., Crab R., Verstraete W. (2010). Primary nutritional content of bio-flocs cultured with different organic carbon sources and salinity. *HAYATI Journal of Biosciences*.

[82] Burford M. A., Thompson P. J., McIntosh R. P., Bauman R. H., Pearson D. C. (2004). The contribution of flocculated material to shrimp (*Penaeus vannamei*) nutrition in a high intensity, zero-exchange system. *Aquaculture*.

[83] Jatobá A., da Silva B. C., da Silva J. S. (2014). Protein levels for *Litopenaeus vannamei* in semi-intensive and biofloc systems. *Aquaculture*.

[84] Gaona C. A. P., Serra F. P., Furtado P. S., Poersch L. H., Wasielesky W. (2016). Biofloc management with different flow rates for solids removal in the *Litopenaeus vannamei* BFT culture system. *Aquaculture International*.

[85] Deb S., Noori M. T., Rao P. S. (2020). Application of biofloc technology for Indian major carp culture (polyculture) along with water quality management. *Aquacultural Engineering*.

[86] Haraz Y. G., Shourbela R. M., El-Hawarry W. N., Mansour A. M., Elblehi S. S. (2023). Performance of juvenile *Oreochromis niloticus* (Nile tilapia) raised in conventional and biofloc technology systems as influenced by probiotic water supplementation. *Aquaculture*.

[87] Shao J., Liu M., Wang B., Jiang K., Wang M., Wang L. (2017). Evaluation of biofloc meal as an ingredient in diets for white shrimp *Litopenaeus vannamei* under practical conditions: effect on growth performance, digestive enzymes and TOR signaling pathway. *Aquaculture*.

[88] Olier B. S., Tubin J. S. B., de Mello G. L., Martínez-Porchas M., Emerenciano M. G. C. (2020). Does vertical substrate could influence the dietary protein level and zootechnical performance of the Pacific white shrimp *Litopenaeus vannamei* reared in a biofloc system?. *Aquaculture International*.

[89] Azim M. E., Little D. C. (2008). The biofloc technology (BFT) in indoor tanks: water quality, biofloc composition, and growth and welfare of Nile tilapia (*O. niloticus*). *Aquaculture*.

[90] Tubin J. S. B., Gutiérrez S. M., Monroy-Dosta M. D. C., Khanjani M. H., Emerenciano M. G. C. (2023). Biofloc technology and cockroach (*Nauphoeta cinerea*) insect meal-based diet for Nile tilapia: zootechnical performance, proximate composition and bacterial profile. *Annals of Animal Science*.

[91] Durigon E. G., Almeida A. P. G., Jerônimo G. T., Baldisserotto B., Emerenciano M. G. C. (2019). Digestive enzymes and parasitology of Nile tilapia juveniles raised in brackish biofloc water and fed with different digestible protein and digestible energy levels. *Aquaculture*.

[92] Sgnaulin T., Pinho S. M., Durigon E. G., Thomas M. C., de Mello G. L., Emerenciano M. G. C. (2021). Culture of pacu *Piaractus mesopotamicus* in biofloc technology (BFT): insights on dietary protein sparing and stomach content. *Aquaculture International*.

[93] Sousa A. A., Pinho S. M., Rombenso A. N., Mello G. L., Emerenciano M. G. C. (2019). Pizzeria by-product: a complementary feed source for Nile tilapia (*Oreochromis niloticus*) raised in biofloc technology?. *Aquaculture*.

[94] Tubin J. B., Paiano D., Hashimoto G. O. (2020). *Tenebrio molitor* meal in diets for Nile tilapia juveniles reared in biofloc system. *Aquaculture*.

[95] Correa A. S., Pinho S. M., Molinari D. (2020). Rearing of Nile tilapia (*Oreochromis niloticus*) juveniles in a biofloc system employing periods of feed deprivation. *Journal of Applied Aquaculture*.

[96] Bauer W., Prentice-Hernandez C., Borges-Tesser M., Wasielesky W., Poersch L. H. S. (2012). Substitution of fishmeal with microbial floc meal and soy protein concentrate in diets for the pacific white shrimp *Litopenaeus vannamei* carnivorous fish. *Aquaculture*.

[97] Valle B. C. S., Dantas E. M., Silva J. F. X. (2015). Replacement of fishmeal by fish protein hydrolysate and biofloc in the diets of *Litopenaeus vannamei* postlarvae. *Aquaculture Nutrition*.

[98] Anand P. S., Kohli M. P. S., Kumar S. (2014). Effect of dietary supplementation of biofloc on growth performance and digestive enzyme activities in *Penaeus monodon*. *Aquaculture*.

[99] Prabu E., Rajagopalsamy C. B. T., Ahilan B., Jeevagan J. M. A., Renuhadevi M. (2018). Effect of dietary supplementation of biofloc meal on growth and survival of GIFT tilapia. *Indian Journal of Fisheries*.

[100] Lunda R., Roy K., Dvorak P., Kouba A., Mraz J. (2020). Recycling biofoc waste as novel protein source for crayfsh with special reference to crayfsh nutritional standards and growth trajectory. *Scientifc Reports*.

[101] Nethaji M., Ahilan B., Kathirvelpandiyan A. (2022). Biofloc meal incorporated diet improves the growth and physiological responses of *Penaeus vannamei*. *Aquaculture International*.

[102] Hersi M. A., Genc E., Pipilos A., Keskin E. (2023). Effects of dietary synbiotics and biofloc meal on the growth, tissue histomorphology, whole-body composition and intestinal microbiota profile of Nile tilapia (*Oreochromis niloticus*) cultured at different salinities. *Aquaculture*.

[103] Dantas E. M., Valle B. C. S., Brito C. M. S., Calazans N. K. F., Peixoto S. R. M., Soares R. B. (2016). Partial replacement of fishmeal with biofloc meal in the diet of postlarvae of the Pacific white shrimp *Litopenaeus vannamei*. *Aquaculture Nutrition*.

[104] Binalshikh-Abubkr T., Hanafiah M. M. (2022). Effect of supplementation of dried bioflocs produced by freeze-drying and oven-drying methods on water quality, growth performance and proximate composition of red hybrid Tilapia. *Journal of Marine Science and Engineering*.

[105] Barzamini B., Harsij M., Adineh H., Jafaryan H. (2021). The effect of biofloc-supplemented diets on the Pacific white shrimp (*Litopenaeus vannamei*): analysis of water quality, growth performance, and biochemical composition. *Iranian Journal of Fisheries Sciences*.

[106] Rani P., Thakur J., Upadhyay A. (2017). Partial replacement of protein using microfloc meal for the diet of mrigal, *Cirrhinus mrigal* fingerlings. *International Journal of Current Microbiology and Applied Sciences*.

[107] Emerenciano M., Ballester E. L. C., Cavalli R. O., Wasielesky W. (2012). Biofloc technology application as a food source in a limited water exchange nursery system for pink shrimp *Farfantepenaeus brasiliensis* (Latreille, 1817). *Aquaculture Research*.

[108] Posadas B. C., Hanson T. R., Leung P. S., Engle C. (2006). Economics of integrating nursery systems into indoor biosecure recirculating saltwater shrimp grow-out systems. *Shrimp Culture: Economics, Market*.

[109] Anand T., Suryakumar B., Nagoormeeran M., Govindraj T. (2015). *Biofloc Technology in Shrimp Culture Systems—Field Experiments Conducted at Hitide Sea Farms, IWASSSS’15*.

[110] Yuan Y., Yuan Y., Dai Y., Gong Y. (2017). Economic profitability of tilapia farming in China. *Aquaculture International*.

[111] Rego M. A. S., Sabbag O. J., Soares R., Peixoto S. (2017). Financial viability of inserting the biofloc technology in a marine shrimp *Litopenaeus vannamei* farm: a case study in the state of Pernambuco, Brazil. *Aquaculture International*.

[112] Rego M. A. S., Sabbag O. J., Soares R. B., Peixoto S. (2017). Risk analysis of the insertion of biofloc technology in a marine shrimp *Litopenaeus vannamei* production in a farm in Pernambuco, Brazil: a case study. *Aquaculture*.

[113] Cang P., Zhang M., Qiao Q. G. (2019). Analysis of growth, nutrition and economic profitability of gibel carp (*Carassius auratus* gibelio ♀ × *Ciprinus carpio* ♂) cultured in zero-water exchange system. *Pakistan Journal of Zoology*.

[114] Panigrahi A., Otta S. K., Kumaraguru Vasagam K. P., Shyne Anand P. S., Biju I. F., Aravind R. (2019). *Training Manual on Biofloc Technology for Nursery and Grow Out Aquaculture*.

[115] Hwihy H. M., Zeina A. F., El-Damhougy K. A. (2021). Influence of biofloc technology on economic evaluation of culturing *Oreochromis niloticus* reared at different stocking densities and feeding rates. *Egyptian Journal of Aquatic Biology & Fisheries*.

[116] Bezerra G. A., Pires D. C., Watanabe A. L., Neto C. C. B., Simões A. R. P., Hisano H. (2022). Economic feasibility and risk analysis of Nile tilapia juveniles reared in a biofloc technology system. *Europe PMC Plus*.

[117] Almeida M. S., Gimenes R. M. T., Furtado P. S. (2022). Economic analysis of intensive and super-intensive *Litopenaeus vannamei* shrimp production in a biofloc technology system. *Boletim do Instituto de Pesca*.

[118] Khanjani M. H., Sajjadi M. M., Alizadeh M., Sourinejad I. (2020). Economic and production evaluation of Pacific white shrimp (*Penaeus vannamei* Boone, 1931) in conventional and biofloc aquaculture systems. *Journal of Animal Environment*.

[119] Khanjani M. H., Alizadeh M. (2021). Biological and economic performance of Nile tilapia (*Oreochromis niloticus*) in two conventional and limited water exchange systems. *Journal of Aquatic Ecology*.

[120] Almeida M. S., Mauad J. R. C., Gimenes R. M. T. (2021). Bioeconomic analysis of the production of marine shrimp in greenhouses using the biofloc technology system. *Aquaculture International*.

[121] Gallardo-Collí A., Pérez-Rostro C. L., Hernández-Vergara M. P. (2019). Reuse of water from biofloc technology for intensive culture of Nile tilapia (*Oreochromis niloticus*): effects on productive performance, organosomatic indices and body composition. *International Aquatic Research*.

[122] Mahanand S. S., Moulick S., Rao P. S. (2013). Water quality and growth of rohu, *Labeo rohita*, in a biofloc system. *Journal of Applied Aquaculture*.

[123] Prates E., Holanda M., Pedrosa V. F., Monserrat J. M., Wasielesky W. (2023). Compensatory growth and energy reserves changes in the Pacific white shrimp (*Litopenaeus vannamei*) reared in different temperatures and under feed restriction in biofloc technology system (BFT). *Aquaculture*.

[124] Huang H.-H., Li C.-Y., Liang T., Lei Y.-J., Yang P.-H., Wu M.-X. (2022). Effects of carbon-to-nitrogen ratio (C : N) on water quality and growth performance of *Litopenaeus vannamei* (Boone, 1931) in the biofloc system with a salinity of 5‰. *Aquaculture Research*.

[125] Khanjani M. H., Alizadeh M., Sharifinia M. (2021). Effects of different carbon sources on water quality, biofloc quality, and growth performance of Nile tilapia (*Oreochromis niloticus*) fingerlings in a heterotrophic culture system. *Aquaculture International*.

[126] Kuhn D. D., Boardman G. D., Craig S. R., Flick G. J., McLean E. (2008). Use of microbial flocs generated from tilapia effluent as a nutritional supplement for shrimp *Penaeus vannamei* in recirculating aquaculture systems. *Journal of the World Aquaculture Society*.

[127] Ray J. A., Lewis B. L., Browdy C. L., Lof J. W. (2010). Suspended solids removal to improve shrimp (*Litopenaeus vannamei*) production and an evaluation of a plant-based feed in minimal-exchange, super intensive culture systems. *Aquaculture*.

[128] Hanson T. R., Posadas B. C., Samocha T., Stokes A. D., Losordo T. M., Browdy C. L., Browdy C. L., Jory D. E. (2009). Economic factors critical to the profitability of super-intensive biofloc recirculating shrimp production systems for marine shrimp *Litopenaeus vannamei*. *Rising Tide, Proceedings of the Special Session on Sustainable Shrimp Farming, World Aquaculture 2009*.

[129] Mugwanya M., Dawood M. A. O., Kimera F., Sewilam H. (2021). Biofloc systems for sustainable production of economically important aquatic species: a review. *Sustainability*.

[130] Shang Y. C., Fast A. W., Lester L. J. (1992). Penaeid markets and economics. *Marine Shrimp Culture: Principles and Practices”*.

[131] Luo G., Gao Q., Wang C. (2014). Growth, digestive activity, welfare, and partial cost-effectiveness of genetically improved farmed tilapia (*Oreochromis niloticus*) cultured in a recirculating aquaculture system and an indoor biofloc system. *Aquaculture*.

[132] Hisano H., Parisi J., Cardoso I. L., Ferri G. H., Ferreira P. M. F. (2020). Dietary protein reduction for Nile tilapia fingerlings reared in biofloc technology. *Journal of the World Aquaculture Society*.

[133] Hlordzi V., Kuebutornye F. K. A., Afriyie G. (2020). The use of *Bacillus* species in maintenance of water quality in aquaculture: a review. *Aquaculture Reports*.

[134] Jones E., van Vliet M. T. H., Qadir M., Bierkens M. F. P. (2021). Country-level and gridded wastewater production, collection, treatment and reuse. *Earth System Science Data*.

[135] Perez-Fuentes J. A., Hernandez-Vergara M. P., Perez-Rostro C. I., Fogel I. (2016). C : N ratios affect nitrogen removal and production of Nile tilapia *Oreochromis niloticus* raised in a biofloc system under high density cultivation. *Aquaculture*.

[136] Perez-Fuentes J. A., Perez-Rostro C. I., Hernandez-Vergara M. P. (2013). Pond-reared Malaysian prawn *Macrobrachium rosenbergii* with the biofloc system. *Aquaculture*.

[137] Verdegem M. C. J., Bosma R. H. (2009). Water withdrawal for brackish and inland aquaculture, and options to produce more fish in ponds with present water use. *Water Policy*.

[138] Taniguchi F. (2010). *Análise de viabilidade técnico-econômica da produção de juvenis de tilápia, Oreochromis niloticus, um estudo de caso*.

[139] Lima E. C. R., Souza R. L., Girao P. J. M., Braga I. F. M., Correira E. S. (2018a). Culture of Nile tilapia in a biofloc system with different sources of carbon. *Revista Ciencia Agronomica*.

[140] Zemor J. C., Wasielesky W., Foes G. K., Poersch L. H. (2019). The use of clarifiers to remove and control the total suspended solids in large-scale ponds for production of *Litopenaeus vannamei* in a biofloc system. *Aquacultural Engineering*.

[141] De Schryver P., Crab R., Defoirdt T., Boon N., Verstraete W. (2008). The basics of bio-flocs technology: the added value for aquaculture. *Aquaculture*.

[142] Dauda A. B., Romano N., Ebrahimi M. (2018). Influence of carbon/nitrogen ratios on biofloc production and biochemical composition and subsequent effects on the growth, physiological status and disease resistance of African catfish (*Clarias gariepinus*) cultured in glycerol-based biofloc systems. *Aquaculture*.

[143] Khanjani M. H., Alizadeh M., Mohammadi M., Sarsangi H. A. (2021c). The effect of adding molasses in different times on performance of Nile tilapia (*Oreochromis niloticus*) raised in a low-salinity biofloc system. *Annals of Animal Scienses*.

[144] Vargas-Albores F., Martínez-Córdova L. R., Gollas-Galván T. (2019). Inferring the functional properties of bacterial communities in shrimp-culture bioflocs produced with amaranth and wheat seeds as fouler promoters. *Aquaculture*.

[145] Rajeev R., Adithya K. K., Kiran S., Selvin J. (2021). Healthy microbiome: a key to successful and sustainable shrimp aquaculture. *Reviews in Aquaculture*.

[146] Bossier P., Ekasari J. (2017). Biofloc technology application in aquaculture to support sustainable development goals. *Microbial Biotechnology*.

[147] Wasielesky W., Krummenauer D., Lara G., Fóes G., Poersch L. (2016). Cultivo de camarões marinhos em sistema de bioflocos: doze anos de pesquisa e desenvolvimento tecnológico na Universidade Federal do Rio Grande – FURG, RS. *Aquaculture Brasil*.

[148] Krummenauer D., Peixoto S., Cavalli R. O., Poersch L. H., Wasielesky W. (2011). Superintensive culture of white shrimp, *Litopenaeus vannamei*, in a biofloc technology system in southern Brazil at different stocking densities. *Journal of the World Aquaculture Society*.

[149] Costa C., Fóes G., Wasielesky W., Poersch L. H. (2018). Different densities in whiteleg shrimp culture using bioflocs and well water in subtropical climate. *Boletim do Instituto de Pesca*.

[150] Nguyen T. A. T., Nguyen K. A. T., Jolly C. (2019). Is super-intensification the solution to shrimp production and export sustainability?. *Sustainability*.

[151] Poersch L. H., Almeida M. S., Gaona C. A., Furtado P., Fóes G., Wasielesky W. (2012). Bioflocos: uma alternativa econômica viável para produtores de camarões em viveiros. *Panorama da Aquicultura*.

[152] Krummenauer D., Júnior C. A. S., Poersch L. H., Foes G. K., de Lara G. R., Wasielesky W. (2012). Cultivo de camarões marinhos em sistema de bioflocos: análise da reutilização da água. *Atlântica (Rio Grande)*.

[153] Shinji J., Nohara S., Yagi N., Wilder M. (2019). Bio-economic analysis of super-intensive closed shrimp farming and improvement of management plans: a case study in Japan. *Fisheries Science*.

[154] Vieira R., Barreto L., Fonseca K., Lordêlo M., de Souza F. R., Evangelista-Barreto N. S. (2019). Zootechnical performance evaluation of the use of biofloc technology in Nile tilapia fingerling production at different densities. *Boletim do Instituto de Pesca*.

[155] Nazarpour S., Mohammadiazarm H. (2023). Optimizing stocking density in biofloc culture of juvenile common carp (*Cyprinus carpio*) using growth and immune-biochemical indices as indicators. *Aquaculture Studies*.

[156] Jackson C. J., Wang Y.-G. (1998). Modelling growth rate of *Penaeus monodon Fabricius* in intensively managed ponds: effects of temperature, pond age and stocking density. *Aquaculture Research*.

[157] Mauladani S., Rahmawati A. I., Absirin M. F. (2020). Economic feasibility study of *Litopenaeus vannamei* shrimp farming: nanobubble investment in increasing harvest productivity. *Jurnal Akuakultur Indonesia*.

[158] Browdy C. L., Bratvold D., Stokes A. D., Mcintosh R. P., Jory E. D., Browdy C. L. (2001). Perspectives on the application of closed shrimp culture systems. *The New Wave, Proceedings of the Special Session on Sustainable Shrimp Culture*.

[159] FAO (2016). *The State of World Fisheries and Aquaculture 2016: Contributing to Food Security and Nutrition for All*.

[160] Teixeira A. P., Guerrelhas A. C. B. (2011). Cultivo Intensivo pode ser a solução para o aumento da produção da carcinicultura?. *Panorama da Aquicultura*.

[161] Tailly J. B. D. (2019). Study of an economical shrimp farming protocol aiming at improving control over water quality. *Mémoire de Fin d’Études d’Ingénieur de l’Institut Supérieur des Sciences agronomiques, agroalimentaires, horticoles et du paysage*.

[162] Kumar G., Engle C. (2017). Economic of intensively aerated catfish ponds. *Journal of the World Aquaculture Society*.

[163] Kumar G., Engle C., Hegde S., Senten J. (2020). Economics of U.S. catfish farming practices: profitability, economies of size, and liquidity. *Journal of the World Aquaculture Society*.

[164] Erondu E., Bekibele D., Gbulubo A. (2006). Optimum crude protein requirement of cat fish, *Chrysichthys nigrodigitatus*. *Journal of Fisheries International*.

[165] Khanjani M. H., Mozanzade M. Torfi, Sharifinia M., Emerenciano M. G. C. (2024). Broodstock and seed production in biofloc technology (BFT): an updated review focused on fish and penaeid shrimp. *Aquaculture*.

[166] Emerenciano M., Cuzon G., Arevalo M., Gaxiola G. (2014). Biofloc technology in intensive broodstock farming of the pink shrimp *Farfantepenaeus duorarum*: spawning performance, biochemical composition and fatty acid profile of eggs. *Aquaculture Research*.

[167] Khanjani M. H. (2021). Reproductive performance of Pacific white shrimp (*Penaeus vannamei* Boone, 1931) broodstocks under the influence of different rearing systems. *Journal of Animal Biology*.

[168] Ekasari J., Zairin M., Putri D. U., Sari N. P., Surawidjaja E. H., Bossier P. (2013). Biofloc-based reproductive performance of Nile tilapia *Oreochromis niloticus* L. broodstock. *Aquaculture Research*.

[169] Cardona E., Lorgeoux B., Chim L., Goguenheim J., Delliou H. L., Cahu C. (2016). Biofloc contribution to antioxidant defence status, lipid nutrition and reproductive performance of broodstock of the shrimp *Litopenaeus stylirostris*: consequences for the quality of eggs and larvae. *Aquaculture*.

[170] Braga A., Lopes D., Magalhães V., Poersch L. H., Wasielesky W. (2015). Use of biofloc technology during the pre-maturation period of *Litopenaeus vannamei* males: effect of feeds with diferente protein levels on the spermatophore and sperm quality. *Aquaculture Research*.

[171] Magana-Gallegos E., Gonzalez-Zuniga R., Arevalo M. (2018). Biofloc and food contribution to grow-out and broodstock of *Farfantepenaeus brasiliensis* (Latreille, 1817) determined by stable isotopes and fatty acids. *Aquaculture Research*.

[172] Alvarenga É. R., de Sales S. C. M., de Brito T. S. (2017). Effects of biofloc technology on reproduction and ovarian recrudescence in Nile tilapia. *Aquaculture Research*.

[173] Manzoor P. S., Rawat K. D., Tiwari V. K., Poojary N., Majeedkutty B. R. Asanaru (2020). Dietary lipid influences gonadal maturation, digestive enzymes and serum biochemical indices of *Cyprinus carpio* reared in biofloc system. *Aquaculture Research*.

[174] Tahoun A. M. (2022). Addition of commercial probiotic (*Lactobacillus delbruekii* and *L. fermentum*) in red tilapia broodstock diet in different rearing systems. I—effects on reproductive performance and larval quality. *Egyptian Journal of Nutrition and Feeds*.

[175] Palacios E., Ibarra A. M., Ramirez J. L., Portillo G., Racotta I. S. (1998). Biochemical composition of eggs and nauplii in white Pacific shrimp, *Penaeus vannamei* (Boone), in relation to the physiological condition of spawners in a commercial hatchery. *Aquaculture Research*.

[176] Luo W., Zhao Y. L., Yao J. J. (2008). Biochemical composition and digestive enzyme activities during the embryonic development of the redclaw crayfish, *Cherax quadricarinatus*. *Crustaceana*.

[177] Pérez-Velasco R., Hernández-Vergara M. P., Pérez-Rostro C., Frías-Quintana C. A. (2023). Variation of dietary protein/lipid levels used in postlarvae of freshwater prawn *Macrobrachium rosenbergii* cultured in a biofloc system. *Latin American Journal of Aquatic Research*.

[178] Cahu C., Guillaume J. C., Stephan G., Chim L. (1994). Influence of phospholipid and highly unsaturated fatty acids on spawning rate and egg and tissue composition in *Penaeus vannamei* fed semi-purified diets. *Aquaculture*.

[179] Xu X. L., Ji W. J., Castell J. D., O’dor R. K. (1994). Influence of dietary lipid sources on fecundity, egg hatchability and fatty acid composition of Chinese prawn (*Penaeus chinensis*) broodstock. *Aquaculture*.

[180] Surai P. F. (2002). *Natural Antioxidants in Avian Nutrition and Reproduction*.

[181] Wabete N., Chim L., Lemaire P., Massabuau J. C. (2005). Caractérisation de problèmes de physiologie respiratoire et d’échanges ioniques associés à la manipulation chez la crevette pénéide *Litopenaeus stylirostris* à 20 C. Styli 2003. *Ifremer, Actes Colloq, Styli*.

[182] Khoshkholgh M. R., Shajani M. Mosapour, Baresari M. Mohammadi (2016). The possibility of partial replacement of olive pomace with some dietary items of rainbow trout (*Oncorhynchus mykiss* Walbaum, 1792). *Journal of Fisheries*.

[183] Bakhshi F., Farah K. Rahmani, Najdegerami E. H., Manaffar R., Tukmachi A. (2018). Chemical and quality indices of common carp meat reared in the biofloc system during the refrigerated storage time. *Journal of Fisheries Science and Technology*.

[184] Najdegerami E. H., Bakhshi F., Lakani F. B. (2016). Effects of biofloc on growth performance, digestive enzyme activities and liver histology of common carp (*Cyprinus carpio* L.) fingerlings in zero-water exchange system. *Fish Physiology Biochemistry*.

[185] Khazaghi M. Abdollahi, Rezaei M., Khazaghi A. Jafarpour (2015). Gel forming and physico-chemical properties of protein recovered from whole and gutted common kilka (*Clupeonella cultriventris*). *Journal of Fisheries Science and Technology*.

[186] Gaona C. A. P., de Almeida M. Souza, Viau V., Poersch L. H., Wasielesky W. (2017). Effect of different total suspended solids levels on a *Litopenaeus vannamei* (Boone, 1931) BFT culture system during biofloc formation. *Aquaculture Research*.

[187] Ebeling J. M., Timmons M. B., Bisogni J. (2006). Engineering analysis of the stoichiometry of photoautotrophic, autotrophic, and heterotrophic removal of ammonia-nitrogen in aquaculture systems. *Aquaculture*.

[188] Holanda M., Santana G., Furtado P. (2020). Evidence of total suspended solids control by *Mugil liza* reared in an integrated system with Pacific white shrimp *Litopenaeus vannamei* using biofloc technology. *Aquaculture Reports*.

[189] Poli M. A., Schveitzer R., Nuñer A. P. O. (2015). The use of biofloc technology in a South American catfish (*Rhamdia quelen*) hatchery: effect of suspended solids in the performance of larvae. *Aquacultural Engineering*.

[190] Pellegrin L., Nitz L. F., Pinto D. D. S. B., Copatti C. E., Wasielesky W., Garcia L. (2022). Effects of suspended solids in the survival and haematological parameters of pacu juveniles (*Piaractus mesopotamicus*) in a biofloc technology culture system. *Aquaculture Research*.

[191] Liu W., Guo Y., Li S., Luo G., Tan H. (2022). The effect of total suspended solids on the nursery of *Penaeus vannamei* nauplius based on biofloc technology system. *Aquaculture Research*.

[192] Carvalho A., Costa L. C., Holanda M., Poersch L. H., Turan G. (2023). Influence of total suspended solids on the growth of the sea lettuce *Ulva lactuca* integrated with the Pacific white shrimp *Litopenaeus vannamei* in a biofloc system. *Fishes*.

[193] Schumann M., Brinker A. (2020). Understanding and managing suspended solids in intensive salmonid aquaculture: a review. *Reviews in Aquaculture*.

[194] Gilmour K. M. (2001). The CO_2_/pH ventilatory drive in fish. *Comparative Biochemistry and Physiology Part A, Molecular & Integrative Physiology*.

[195] Johnson R. A. (2017). A quick reference on respiratory alkalosis. *Veterinary Clinics of North America: Small Animal Practice*.

[196] Palmer B. F. (2012). Evaluation and treatment of respiratory alkalosis. *American Journal of Kidney Diseases*.

[197] Moura P., Neto I. A., Brandão H., Furtado P., Poersch L., Wasielesky W. (2023). Effects of magnesium reduction in artificial low-salinity water on the growth of Pacific white shrimp *Litopenaeus vannamei* in a biofloc system. *Aquaculture*.

[198] Khanjani M. H., Sharifinia M. (2021). Production of Nile tilapia *Oreochromis niloticus* reared in a limited water exchange system: the effect of different light levels. *Aquaculture*.

[199] FAO (2022). *The State of World Fisheries and Aquaculture 2022: Toward Blue Transformation*.

[200] Touiri L., Najjar F. Z., Rolle F., Crespi V. (2020). Aquaculture capacity development in morocco through the establishment of an aquaculture demonstration center for the training of qualified personnel. *FAO Aquaculture Newsletter*.

[201] Seixas S., Saravanan S., Gonçalves S. (2015). Innovation and educational changes: two e-learning cases in aquaculture. *Aquaculture International*.

[202] Lara G., Hostins B., Bezerra A., Poersch L., Wasielesky W. (2017). The effects of different feeding rates and re-feeding of *Litopenaeus vannamei* in a biofloc culture system. *Aquacultural Engineering*.

[203] Liang W., Luo G., Tan H., Ma N., Zhang N., Li L. (2014). Efficiency of biofloc technology in suspended growth reactors treating aquacultural solid under intermittent aeration. *Aquacultural Engineering*.

[204] de Morais A. P. M., Abreu P. C., Wasielesky W., Krummenauer D. (2020). Effect of aeration intensity on the biofilm nitrification process during the production of the white shrimp *Litopenaeus vannamei* (Boone, 1931) in biofloc and clear water systems. *Aquaculture*.

[205] Harun A. A. C., Mohammad N. A. H., Ikhwanuddin M., Jauhari I., Sohaili J., Kasan N. A. (2019). Effect of different aeration units, nitrogen types and inoculum on biofloc formation for improvement of Pacific whiteleg shrimp production. *The Egyptian Journal of Aquatic Research*.

[206] Lim Y. S., Ganesan P., Varman M., Hamad F. A., Krishnasamy S. (2021). Effects of microbubble aeration on water quality and growth performance of *Litopenaeus vannamei* in biofloc system. *Aquacultural Engineering*.

[207] Khanjani M. H., Sharifinia M., Emerenciano M. G. C. (2023). A detailed look at the impacts of biofloc on immunological and hematological parameters and improving resistance to diseases. *Fish and Shellfish Immunology*.

[208] Yacout D. M. M., Soliman N. F., Yacout M. M. (2016). Comparative life cycle assessment (LCA) of tilapia in two production systems: semi-intensive and intensive. *International Journal of Life Cycle Assessment*.

[209] Bohnes F. A., Hauschild M. Z., Schlundt J., Laurent A. (2019). Life cycle assessments of aquaculture systems: a critical review of reported findings with recommendations for policy and system development. *Reviews in Aquaculture*.

